# Cxcr3 promotes protection from colorectal cancer liver metastasis by driving NK cell infiltration and plasticity

**DOI:** 10.1172/JCI184036

**Published:** 2025-04-01

**Authors:** Eleonora Russo, Chiara D’Aquino, Chiara Di Censo, Mattia Laffranchi, Luana Tomaipitinca, Valerio Licursi, Stefano Garofalo, Johann Promeuschel, Giovanna Peruzzi, Francesca Sozio, Anna Kaffke, Cecilia Garlanda, Ulf Panzer, Cristina Limatola, Christian A.J. Vosshenrich, Silvano Sozzani, Giuseppe Sciumè, Angela Santoni, Giovanni Bernardini

**Affiliations:** 1 Department of Molecular Medicine, Laboratory Affiliated to Istituto Pasteur Italia - Fondazione Cenci Bolognetti, Sapienza University of Rome, Rome, Italy.; 2 Innate Immunity Unit, Institut Pasteur, Université Paris Cité, INSERM U1223, Paris, France.; 3 Institute of Molecular Biology and Pathology, National Research Council (IBPM-CNR), Rome, Italy.; 4 Department of Physiology and Pharmacology, Sapienza University of Rome, Rome, Italy.; 5 Center for Life Nano & Neuro Science, Istituto Italiano di Tecnologia, Rome, Italy.; 6 Department of Surgery, Sapienza University of Rome, Rome, Italy.; 7 III. Department of Medicine, University Medical Center Hamburg-Eppendorf, Hamburg, Germany.; 8 Department of Biomedical Sciences, Humanitas University, Via Rita Levi Montalcini 4, 20090 Pieve Emanuele, Milan, Italy.; 9 IRCCS Humanitas Research Hospital, via Manzoni 56, Rozzano, Milan, Italy.; 10 Istituto di Ricovero e Cura a Carattere Scientifico Neuromed, Isernia, Italy.

**Keywords:** Immunology, Oncology, Cell migration/adhesion, Innate immunity, NK cells

## Abstract

The antimetastatic activity of NK cells is well established in several cancer types, but the mechanisms underlying NK cell metastasis infiltration and acquisition of antitumor characteristics remain unclear. Herein, we investigated the cellular and molecular factors required to facilitate the generation of an ILC1-like CD49a^+^ NK cell population within the liver metastasis (LM) environment of colorectal cancer (CRC). We show that CD49a^+^ NK cells had the highest cytotoxic capacity among metastasis-infiltrating NK cells in the MC38 mouse model. Furthermore, the chemokine receptor CXCR3 promoted CD49a^+^ NK cell accumulation and persistence in metastasis where NK cells colocalize with macrophages in CXCL9- and CXCL10-rich areas. By mining a published scRNA-seq dataset of a cohort of patients with CRC who were treatment naive, we confirmed the accumulation of CXCR3^+^NK cells in metastatic samples. Conditional deletion of *Cxcr3* in NKp46^+^ cells and antibody-mediated depletion of metastasis-associated macrophages impaired CD49a^+^NK cell development, indicating that CXCR3 and macrophages contribute to efficient NK cell localization and polarization in LM. Conversely, CXCR3^neg^ NK cells maintained a CD49a^–^ phenotype in metastasis with reduced parenchymal infiltration and tumor killing capacity. Furthermore, CD49a^+^ NK cell accumulation was impaired in an independent SL4-induced CRC metastasis model, which fails to accumulate CXCL9^+^ macrophages. Together, our results highlight a role for CXCR3/ligand axis in promoting macrophage-dependent NK cell accumulation and functional sustenance in CRC LM.

## Abstract 2

The chemokine receptor CXCR3 supports NK cell migration and persistence into colorectal cancer liver metastasis, where NK cells are activated by macrophages and restrain metastasis growth

## Graphical abstract

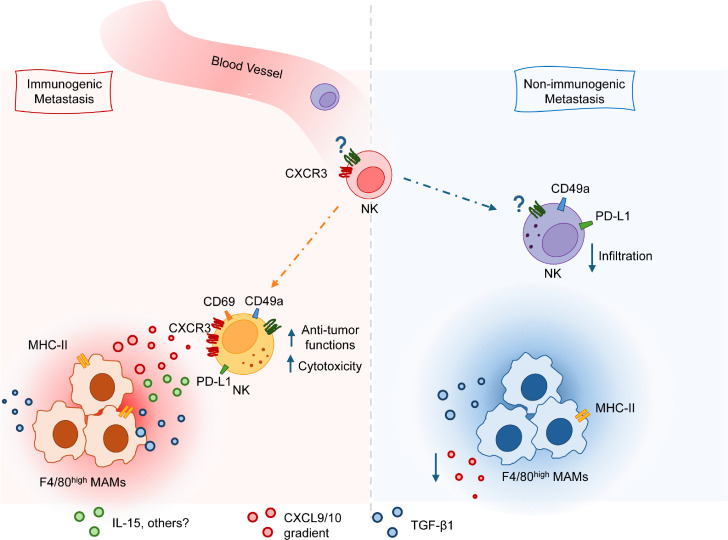

## Introduction

Colorectal cancer (CRC) is the third most frequent cancer and the second cause of cancer-related death world wide. Poor prognosis is mainly associated with metastasis onset, with 70% of metastases being confined to the liver (1).

At steady state, the liver contains conventional NK cells (cNK), which are defined as CD49a^–^CD49b^+^Eomes^+^, and tissue-resident CD49a^+^CD49b^–^Hobit^+^ type 1 innate lymphoid cells (ILC1s) (2, 3). NK cells are cytotoxic lymphocytes exerting key functions in protection against cancer initiation and metastasis and serve as prognostic factors in several cancer types (4, 5). Recent studies revealed that ILC1 subsets express granzymes and show killing capacity, challenging their traditional classifications as helper-like cells (6, 7) and indicating the functional competence of liver innate lymphocytes to respond to and eliminate disseminated tumor cells. Indeed, a recent study by Ducimetière et al. found that ILC1s are necessary in early stages to inhibit CRC liver metastatic seeding, while NK cells have enduring antimetastatic functions in MC38-injected mice (8). This function of NK cells may be acquired following activation by liver macrophages, which can promote NK cell tumoricidal function by secreting IL-18 or presenting 41BBL (9, 10). Conversely, an additional ILC state has been identified within the metastatic environment, resembling a CD49a^+^ ILC subset previously observed in various pathological and tumoral conditions, resulting from NK cell conversion induced by TGF-β1 (8, 11, 12). The role of these ILC1-like cells in cancer is controversial, with opposing functional outcomes such as immune suppression or potent effector functions, possibly linked to different tumor contexts (11, 13, 14). Hence, understanding the mechanisms that enable NK cells to access local metastatic niches, thereby contributing to their plasticity, will facilitate the identification of key immune regulators that critically affect metastatic tumor progression. In this regard, chemotactic factors and adhesion molecules deeply influence the evolution of tumor microenvironment (TME) by directing immune cell infiltration and retention (15).

Chemokine receptor expression drives distinct migratory patterns in NK cell subsets with diverse effector functions, enabling their tissue localization during immune surveillance or pathological conditions (16–19).

The chemokine receptor CXCR3 has been shown to promote antitumor response by favoring T and NK cell tumor infiltration (18, 20–22), but there is no evidence on its impact on NK cell behavior in the metastasis microenvironment (MME). Furthermore, CXCL9 expression levels are positively correlated with overall survival and disease-free survival in CRC (23, 24), although the underlying mechanism is still unclear.

Here, to understand the impact of the specific MME on the development and behavior of type 1 innate lymphocytes, we employed 2 distinct orthotopic mouse models of liver metastasis based on intrasplenic injection of murine colon adenocarcinoma cell lines characterized by contrasting mutational status of mismatch repair genes. Transcriptomic and FACS analyses show abundant infiltration of a CD49a^+^CD49b^+^ILC1-like NK cell population, which displays features of proliferation, active glycolysis, and tissue residency, as well as of activation by IL-15 and type I interferon in MC38-induced metastasis. Conversely, CD49a^+^NK cell tumor infiltration was profoundly impaired in SL4-induced metastasis. We showed that metastasis-associated macrophages (MAMs) are important regulators of NK cell plasticity, able to promote the ILC1-like phenotype in vitro and in vivo. Finally, by generating a mouse model with NKp46^+^ ILC-specific deletion of *Cxcr3* gene, we demonstrated a key role of CXCR3 in CD49a^+^ILC1-like NK cell differentiation in vivo and that CXCR3^+^ NKp46^+^ cells restrain liver metastasis outgrowth.

In summary, our data reveal a nonredundant role of CXCR3 in regulating ILC1-like NK cell accumulation in metastasis, linked to the ability of this receptor to facilitate NK cell activation by macrophages in liver metastasis.

## Results

### The MC38 liver MME induces generation of NK cells with proliferative and tissue-residency transcriptional profiles marked by CD49a expression.

**Figure 1:**
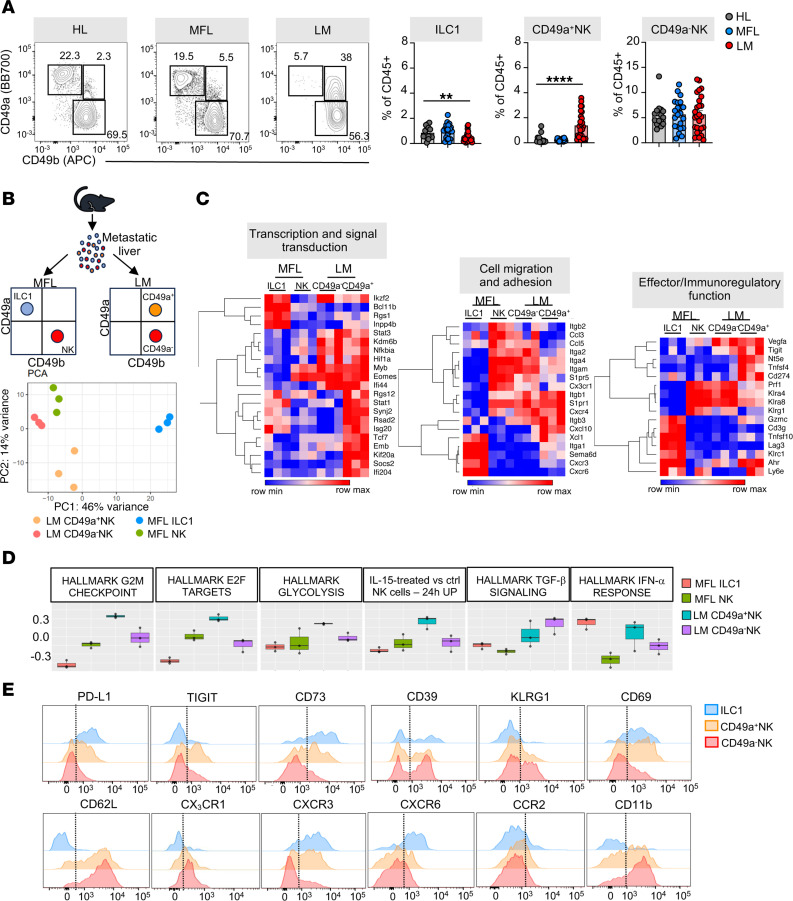
The liver metastasis microenvironment reorganizes type 1 ILC compartment and deeply shapes their transcriptional profile. (**A**) Left, representative contour plots of ILC1s (CD49a^+^CD49b^–^), CD49a^–^NK cells (CD49a^–^CD49b^+^) and CD49a^+^NK cells (CD49a^+^CD49b^+^) in healthy liver (HL), metastasis-free liver (MFL), and liver metastasis (LM) gated on lin(CD3CD19)^–^CD45^+^NK1.1^+^ NKp46^+^ cells. Right, histograms show mean ± SEM of ILC1, CD49a^+^ NK, and CD49a^–^ NK cell frequency among CD45^+^ cells in HL, MFL, and LM of MC38-injected tumor-bearing mice. 5 independent experiments with at least 5 mice per group were performed. (*n* ≥ 15, ILC1 **P = 0.007; CD49a^+^NK ****P < 0.0001, 1-way ANOVA). (**B**) Top, gating scheme for cell populations isolated by fluorescence activated cell sorting. Pooled MFL and LM samples from at least 6 mice were collected in 3 independent sortings. Bottom, principal component analysis (PCA) from bulk RNA-seq. (**C**) Heatmaps showing DEGs involved in regulation of cellular functions. (**D**) Gene set variation analysis (GSVA) of MFL ILC1 and NK cells and LM CD49a^–^ NK cells and CD49a^+^ NK showing modulation of pathways associated to cell proliferation (E2F targets, G2M checkpoint), activation (IL-15-treated NK cells, TGF-β1, and IFN-α signalling) and metabolism (glycolysis). (**E**) Representative FACS histogram plots of at least 3 independent analyses for cell surface receptor expression by LM CD49a^+^ NK cells, ILC1, and CD49a^–^ NK cells.

NK cells and ILC1 robustly participate in the protection against MC38 cell seeding and growth in the liver (8, 9). Nevertheless, the mechanisms involved in shaping new NK cell phenotypes in the MME and their relevance in the antitumor immune response remain unclear. In the MC38 model, we found that liver metastasis growth was associated with the expansion of a CD49a^+^CD49b^+^ NK cell subset and with marked NK cell infiltration, while ILC1s were almost excluded from liver metastasis (LM) (Figure 1A). Transcriptomic analysis of sorted type 1 ILC populations from MC38 metastasis (Supplemental Figure 1A; supplemental material available online with this article; https://doi.org/10.1172/JCI184036DS1) revealed that CD49a^+^ NK cells clustered close to CD49a^–^ NK cells from metastasis-free liver (MFL) and LM and not to MFL ILC1 (Figure 1B). However, compared to NK cells, they exhibited increased expression of genes that characterize liver ILC1s, including *Itga1*, *Tnfsf10*, *Ahr*, *Socs2*, *Inpp4b*, *Gzmc*, and *Sema6d* (25). CD49a^+^ NK cells showed low expression of *Itga4*, *Itgam*, *Itga2*, *S1pr5*, and *Cx3cr1*, as well as the Ly49 receptors *Klra4* and *Klra8*, which are expressed by conventional NK cells but not by ILC1. Furthermore, CD49a^+^NK cells displayed upregulated expression of the transcription factor *Tcf7* in respect to CD49a^–^ NK cells, while *Bcl11b* was downmodulated, suggesting an immature phenotype with a program of cell survival and proliferation (Figure 1C and Supplemental Figure 1B) (26, 27). Applying gene expression signatures from the molecular signature database (MSigDB) to the transcriptional profile of ILCs, we found that CD49a^+^NK cells exhibited higher expression of genes linked to cell proliferation, IFN-α response, and IL-15 activation, compared with CD49a^–^ NK cells from MFL and LM, while the TGF-β signaling pathway was upregulated in both CD49a^–^ and CD49a^+^ NK cells from LM relative to MFL NK cells, suggesting an involvement of these pathways in CD49a^+^ NK cell accumulation and functional adaptation (Figure 1D).

CD49a^+^ NK cells robustly modulated their adhesion and chemotactic receptor expression pattern showing induced expression of *Itgb3*, *Itga1*, and *Ccr5* and downmodulation of *Itga2*, *Itga4*, *Itgam*, *S1pr5*, and *Cx3cr1* compared with NK cells (Figure 1C). This latter observation suggests that recently recruited NK cells undergo transcriptional changes to enhance their retention into metastasis as previously demonstrated in a s.c. tumor mouse MC38 model (28). Gene Ontology (GO) enrichment analysis further indicated that CD49a^+^NK cells are more enriched in pathways involving positive regulation of cell-cell adhesion and cell differentiation, upregulate genes involved in mitosis compared with LM NK cells, and also express lower levels of genes involved in leukocyte chemotaxis and migration (Supplemental Figure 1C).

We applied our bulk RNA-seq–derived signatures to NK clusters obtained from a previously published single-cell transcriptomic dataset of MC38-induced liver metastasis (8). We found that single cell clusters NK_a, NK_b, and NK_e exhibited higher similarity to the LM CD49a^–^NK cell signature, whereas clusters NK_c, NK_d, and NK_f displayed a higher score for CD49a^+^ NK cell signature (Supplemental Figure 2A). To follow the changes of NK cell states occurring in transdifferentiating NK cells, we performed a pseudotime analysis. The resulting data indicated that NK cells that recently infiltrate the metastasis undergo transcriptional reprogramming to acquire features of the NK_c cluster, which downmodulates expression of genes involved in lymphocyte recirculation (*Klf2* and *Klf6*) and in NK cell activation and maturation (*Fos, Jun,* and *Zeb2*). The NK_c cluster can progress either toward a NK cell state (NK_b) or toward a tissue resident ILC1 phenotypes (trILC_a, trILC_b) through cluster NK_f, corresponding to a transitional CD49a^+^NK phenotype (Supplemental Figure 2B).

Differentially expressed genes (DEGs) analysis revealed upregulation of several genes involved in NK cell activation and tissue retention (i.e., *Cd160, Itga1, Rgs1,* and *Cd7*) and ILC1 characteristic genes (*Cd7*, *Ly6e*, *Tnfsf10*, *Cxcr3*, and *Xcl1*) as NK cells transit from NK_c to the NK_f state, while other genes involved in NK cell maturation and homing (i.e., *Irf8, Zeb2, Prf1,* and *Klf2*) were upregulated during transition to cluster NK_b (Supplemental Figure 2, C and D).

We conclude that type-1 innate lymphocytes in metastasis comprehend NK cell state transitions dependent on local signals in the evolving environment. Indeed, a relevant fraction of NK cells preserve a phenotype very close to MFL cNK cells, while NK cell transdifferentiation into CD49a^+^ NK cells is associated with acquisition of higher proliferative and tissue residency state and ILC1 genes.

### CXCR3 is prevalently expressed by CD49a^+^NK cell subsets in mouse and human liver metastasis.

**Figure 2:**
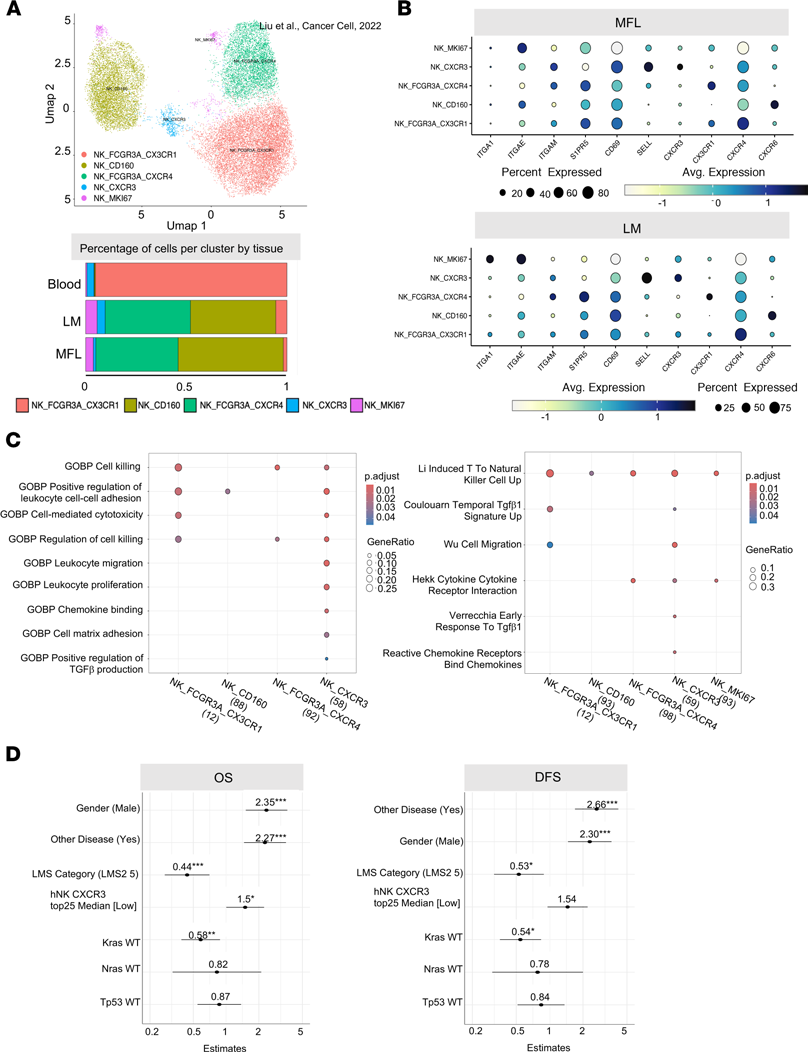
CXCR3 is prevalently expressed by CD49a^+^ NK cell subsets in human liver metastasis. (**A**) Top, Umap showing 5 NK cell clusters from MFL, LM, and PBMC samples identified in the analysis of human CRC dataset from Liu et al. (32). Bottom, frequency of NK cell clusters in MFL, LM, and PBMCs. (**B**) Dot plots showing gene expression related to cell migration and adhesion in NK cell clusters from MFL and LM. (**C**) GO analysis in NK cell clusters. (**D**) Forest plot analysis showing overall survival (OS) and disease-free survival (DSF) of NK_CXCR3 signature applied on colorectal cancer liver metastasis dataset (33).

The role of CD49a^+^NK cells remains contentious (8, 11, 28, 29). Therefore, we aimed to further investigate the phenotypic and functional characteristics of LM CD49a^+^NK cells. CD49a^+^NK cells exhibit higher surface expression levels of the immune checkpoint (IC) molecules PD-L1 and Tigit as well as CD73, while displaying lower levels of KLRG1 expression compared with LM CD49^–^ NK cells, consistent with an ILC1-like and/or an activated NK cell phenotype (30, 31). In contrast, both LM CD49^–^ and CD49a^+^ NK cells show lower expression of CD62L and CX3CR1 compared with MFL NK cells (Figure 1E and Supplemental Figure 3, A and B) and were highly proliferating in vivo, as determined by Ki-67 and BrdU incorporation staining (Supplemental Figure 3, C and D). Furthermore, higher expression levels of the homing receptor CXCR3 and of the tissue retention marker CD69 suggest that CD49a^+^NK cells represent NK cells with a selective advantage in the ability to extravasate and persist in the metastasis parenchyma. These genes were also enriched in NK cell populations mined from a published scRNA-seq dataset of liver metastasis from a cohort of patients with CRC, validating the translational relevance of this observation (32); we found higher frequency of NK_FCGR3A_CXCR4, NK_CXCR3 and NK_MKI67 clusters in LM compared with MFL (Figure 2A and Supplemental Figure 4A), as NK_CXCR3 was enriched in transcripts linked to immature states (i.e., CCR7, TCF7, IL7R and SELL), while NK_MKI67 was for genes associated with survival and proliferation (i.e., MYC, BCL2 and CD9). NK_MKI67 and NK_CXCR3 were also characterized by coexpression of *CXCR3* and residency markers (i.e. *CD69, ITGA1,* and *ITGAE*), and reduced *S1PR5* expression levels, indicating enhanced tissue retention features that may depend on CXCR3 expression (Figure 2B, Table 1, and Supplemental Information on Figure 2 dataset). GO analysis showed activation of pathways linked to cytotoxicity in NK_CXCR3 and the 2 NK_FCGR3A clusters and highlights NK_CXCR3’s adhesion, migration, and proliferation programs in metastases (Figure 2C). Prompted by this observation, we derived the NK_CXCR3 signature from the top 25 expressed genes and assessed its prognostic value in colorectal cancer liver metastases using a publicly available microarray dataset (33). Multiparametric Cox regression analysis showed that lower NK_CXCR3 representation correlated with higher mortality risk and other prognostic factors (Figure 2D).

These findings suggest that accumulation in the metastatic liver of NK cell subsets expressing *CXCR3* and *CD49a* and exhibiting features associated with tissue residency and low maturation correlates with a better prognosis in patients with CRC.

### NK cell liver metastasis infiltration depends on CXCR3.

**Figure 3:**
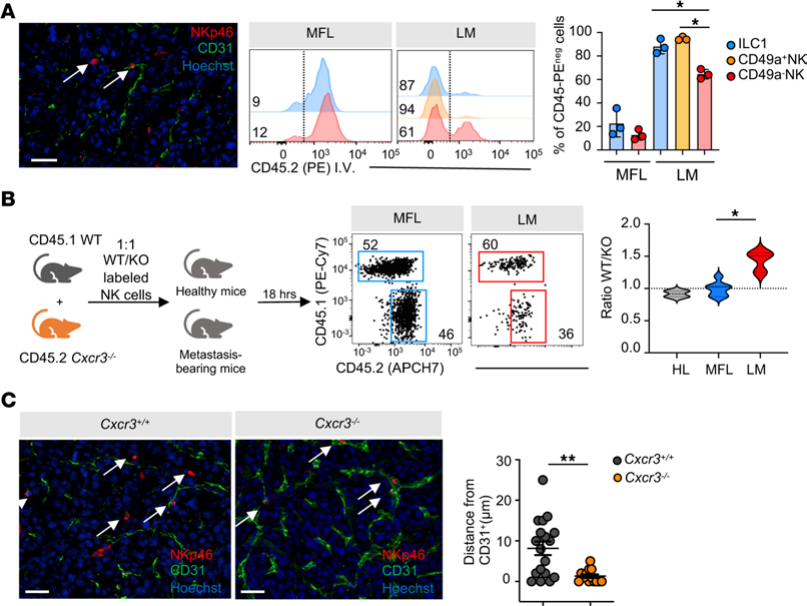
CXCR3 chemokine receptor drives NK cell infiltration in liver metastasis. (**A**) Left, representative immunofluorescence image of NKp46^+^ cell localization relative to endothelial (CD31^+^) cells (*n* = 4 mice, scale bar: 50 μm). Right, representative FACS histogram plots and quantification of parenchymal localization of ILC1, CD49a^+^ NK and CD49a^–^ NK cells assessed by CD45 staining upon CD45-PE in vivo injection before mouse sacrifice in MFL and LM. Numbers in FACS plots represent frequency of CD45-PE^–^ cells. Histogram bars show mean ± SD of CD45-PE^–^ cell frequency among cell populations in a representative experiment (ILC1 *P = 0.02, CD49a^+^ NK *P = 0.014, 1-way ANOVA) out of 3 performed with a total of 8 mice. (**B**) Schematics of NK cell competitive adoptive transfer experiments. CFSE^+^ splenic NK cells from CD45.1 WT and CD45.2 *Cxcr3^–/–^* mice were i.v. transferred in healthy control or metastasis-bearing mice at 1:1 ratio. Transferred cells were identified as CFSE^+^ cells. WT and *Cxcr3^–/–^* cells were discriminated according to their CD45 allelic variant. Violin plot shows the relative number of NK cells from WT and *Cxcr3^–/–^* mice, expressed as WT/KO ratio of transferred cells in HL, MFL, and LM in 2 independent experiments (HL *n* = 2, MFL *n* = 7, and LM *n* = 3; *P = 0.01, 1-way ANOVA). (**C**) Representative immunofluorescence staining of LM from 4 mice; in red, NKp46^+^ cells are indicated by white arrows and CD31^+^ blood vessels are shown in green. Relative quantification of NKp46^+^ cells distance from blood vessels in LM of *Cxcr3^+/+^* and *Cxcr3^–/–^* mice (**P = 0.0018 2-tailed Student’s *t* test; scale bar: 50μm).

To understand to what extent type 1 ILCs infiltrate the metastasis parenchyma, we analyzed the location of these cells in the metastatic nodules. In metastasis sections, NKp46^+^ cells were found mostly close to the vasculature, identified by CD31 staining (Figure 3A, left). To determine the intrametastasic distribution of different type 1 ILCs in metastasis, we performed in vivo labeling using a CD45-PE mAb which selectively stains vascular-associated cells. In MFL, most NK cells and ILC1 were stained, confirming their typical distribution in sinusoids (2). In LM, a relevant fraction of CD49a^–^CD49b^+^NK cells stained positive, indicating a vascular location, ILC1 and CD49a^+^CD49b^+^NK cells were mostly CD45-PE^–^, demonstrating a higher capacity to infiltrate the metastasis parenchyma (Figure 3A, center and right).

The acquisition of the ILC1-like cell phenotype by CD49a^+^NK cells is closely associated with CXCR3 expression (Figure 1E), prompting us to investigate whether NK cell infiltration in the metastasis is CXCR3 dependent. To investigate this, we mixed at a 1:1 ratio CFSE-labeled splenic CD3^–^ NK1.1^+^ cells purified from CD45.1 *WT* and CD45.2 *Cxcr3^–/–^* mice and injected them into healthy and metastasis-bearing mice. After 18 hours, the livers of recipient mice were assessed for accumulation of donor NK cells. *Cxcr3*-deficient NK1.1^+^ cells displayed a 50% reduced capacity of entry into the metastasis of recipient mice compared with WT NK cells, whereas they migrated similarly to WT NK cells into the HL and MFL (Figure 3B).

Immunofluorescence staining of tissue section from tumor-bearing mice revealed NKp46^+^ cells infiltrating the metastasis in Cxcr3^+/+^ mice, whereas these cells were predominantly associated with vessels in Cxcr3^–/–^ mice, further supporting CXCR3’s role in NKp46^+^ cell extravasation and tumor infiltration (Figure 3C).

As a result, we concluded that CXCR3 marks NK cell populations with tissue-resident features that preferentially accumulate in the metastases of CRC patients and mouse models and is required for efficient NK cell metastasis infiltration.

### CXCR3-expressing CD49a^+^ NK cells preferentially localize in activating metastatic niches.

**Figure 4:**
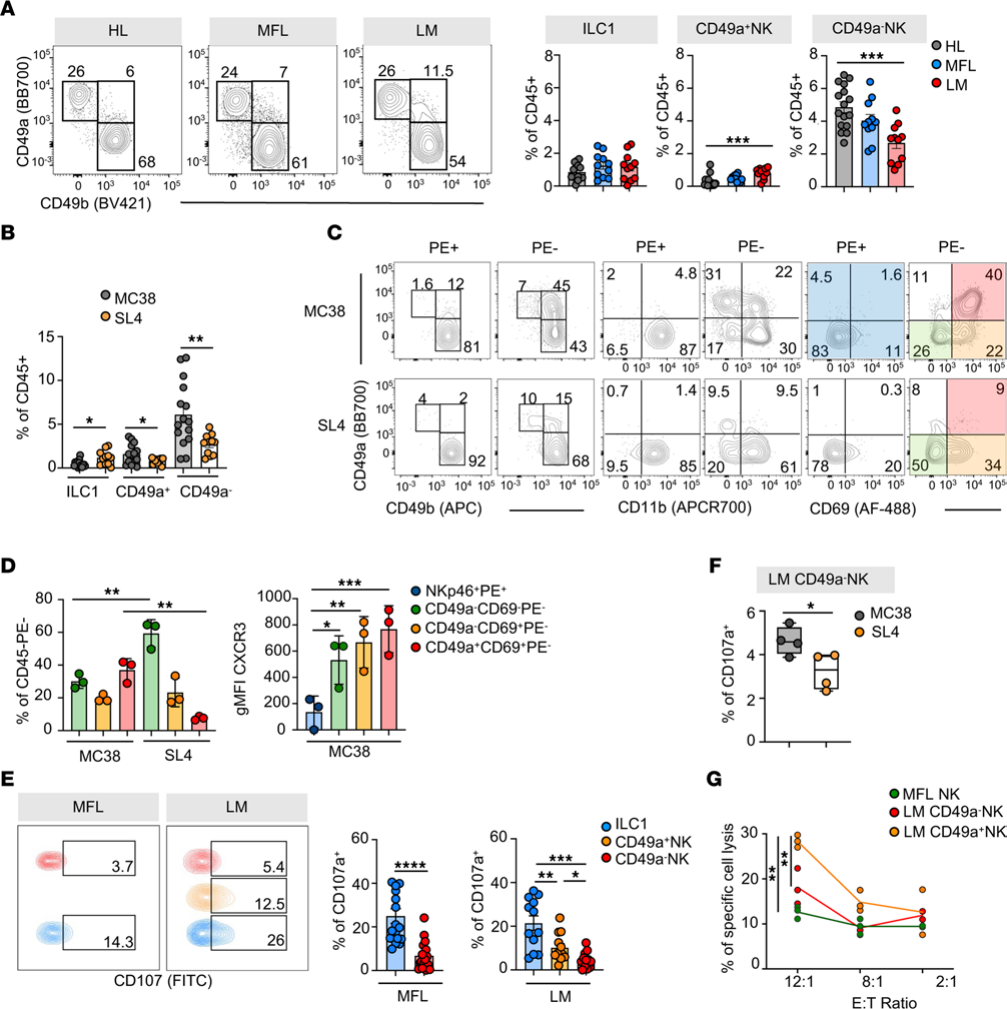
CD49a^+^ NK cells accumulate in metastasis parenchyma and acquire features of tissue residency in MC38- but not in SL4-induced metastasis. (**A**) Left, representative contour plots of type 1 innate lymphocytes in HL, MFL, and LM of SL4-injected mice gated on lin(CD3CD19)^–^CD45^+^NK1.1^+^ NKp46^+^ cells. Right, histograms show mean ± SEM of ILC1, CD49a^+^ and CD49a^–^ NK cell frequency among CD45^+^ cells. 3 independent experiments with at least 11 total mice (CD49a^+^ NK ***P = 0.0008, CD49a^–^ NK ***P = 0.0002 2-tailed Student’s *t* test). (**B**) Histogram plot shows comparison of mean frequency ± SEM of cells among CD45^+^ cells in LM from MC38- and SL4-injected mice. (ILC1 *P = 0.02, CD49a^+^ NK *P = 0.04, CD49a^–^ NK **P = 0.006 2-tailed Student’s *t* test). (**C**) Left, representative contour plots of ILC1, CD49a^+^ and CD49a^–^ NK cell distribution in vascular (CD45-PE^+^) and parenchymal (CD45-PE^–^) compartments of MC38- and SL4-induced LM upon CD45-PE in vivo labeling. Middle and right, CD49a, CD11b, and CD69 expression on total CD49b^+^ cells among CD45-PE^+^ and CD45-PE^–^ cells. (**D**) Left, histogram plot shows frequency of CD45-PE^–^ cells in LM from MC38- and SL4-injected mice (CD49a^–^CD69^–^ **P = 0.001, CD49a^+^CD69^+^ **P = 0.001 1-way ANOVA). Right, histogram plot shows a representative experiment of CXCR3 geometric (g) MFI ± SD on NK cell subsets in MC38-induced LM. Three animals per group were analyzed (CD49a^–^CD69^–^ *P = 0.017, CD49a^–^CD69^+^ **P = 0.0018, CD49a^+^CD69^+^ ***P = 0.0004, 1-way ANOVA). (**E**) Contour plots show CD107a expression by MC38-induced MFL and LM type 1 innate lymphocytes. Histograms show mean frequency ± SEM of CD107a^+^ cells from 3 independent experiments (MFL: *n* = 18 ****P < 0.0001, LM: *n* = 8 CD49a^+^ NK *P = 0.03, LM CD49a^–^ NK *P = 0.01, 1-way ANOVA). (**F**) Box plot showing frequency of CD107a^+^ LM CD49a^–^ NK cells in MC38- and SL4-derived LM (*n* = 4 *P = 0.03 2-tailed Student’s *t* test). (**G**) Representative experiment out of 3 (in duplicate) showing mean frequency ± SD of MC38 target cell–specific lysis by sorted MFL NK cells and LM CD49a^+^ and CD49a^–^ NK cells.

To assess if CD49a^+^NK cell accumulation in liver metastasis may be specifically linked to the immunogenic microenvironment induced by MC38 cells (34), we evaluated type 1 ILC metastasis distribution in an independent mouse model characterized by lower immune cell infiltration (35). SL4-induced metastases displayed a milder CD49a^–^ and CD49a^+^ NK cell infiltration, while ILC1 cells readily infiltrated the metastasis compared with MC38-induced metastasis (Figure 4, A and B and Supplemental Figure 5A).

Similar to MC38 metastasis, CD49a^–^ but not CD49a^+^ NK cells were labeled with CD45-PE in SL4-induced metastasis. We focused on the CD49b^+^ cell fraction to compare the maturation state and tissue retention features of CD49a^–^ and CD49a^+^ NK cells infiltrating the metastasis parenchyma (i.e., the CD45-PE^neg^ cell fraction), by analyzing CD11b and CD69 expression, respectively. In MC38-induced metastasis, CD11b was expressed by most NK cells and by a relevant portion of CD49a^+^ NK cells. CD69 was expressed by a fraction of CD49a^–^ NK cells and by most CD49a^+^ NK cells, suggesting that CD49a expression is induced on NK cells that have already gained tissue residency (Figure 4C, upper). Conversely, in SL4-induced metastasis, CD11b and CD69 were not expressed by CD49a^+^ NK cells, pointing to a phenotype of immature and recently infiltrating cells (Figure 4C, lower and Figure 4D, left). Moreover, while intravascular CD45-PE^+^NK cells poorly express CXCR3, CD45-PE^–^CD69^+^ cells show the highest levels of CXCR3 expression, regardless of CD49a expression (Figure 4D, right). This suggests that CXCR3 is involved in NK cell infiltration into metastasis and their localization in tissue niches required for generation and tissue persistence of CD49a^+^ NK cells. To establish if stronger tissue residency traits of CD49a^+^ NK cells from MC38-derived liver metastasis could be mirrored by higher functional properties, we analyzed differences in effector functions among LM NK cell subsets. We found that LM CD49a^–^ and CD49a^+^ NK cells secreted similar amounts of IFN-γ at higher levels than NK cells from HL and MFL (Supplemental Figure 5B), and that ILC1 displayed the higher degranulation capacity, followed by CD49a^+^NK cells (Figure 4E). Parallel experiments to compare NK cell function in MC38 and SL4-induced metastasis, demonstrated lower degranulation capacity in CD49a^–^NK cells from SL4-induced metastasis and no differences in ILC1 and CD49a^+^NK cell degranulation (Figure 4F and data not shown). Furthermore, CD49a^+^NK cells acquired granzyme C expression in MC38-, but not in SL4-derived LM (Supplemental Figure 5, C and D).

To understand whether higher degranulation capacity and granzyme C expression corresponded to a higher killing ability, we performed an in vitro cytotoxicity assay of sorted CD49a^–^ and CD49a^+^NK cell populations from MC38-induced MFL and LM. The results indicate that CD49a^+^NK cells have a prominent killing capacity in the metastatic environment, in line enriched pathways associated with cell cytotoxicity in the human NK cell cluster expressing *ITGA1* (Figure 4G and Figure 2C).

In conclusion, CD49a^+^NK cells accumulating in MC38-induced but not SL4-induced metastasis display a tissue-resident phenotype that potentially sustains effector functions and shows features of activated cells, such as CD69, Tigit, and PD-L1 expression and enhanced cytotoxicity.

### Two main macrophage populations infiltrate MC38-induced LM, which differ in maturation and functional marker expression.

**Figure 5:**
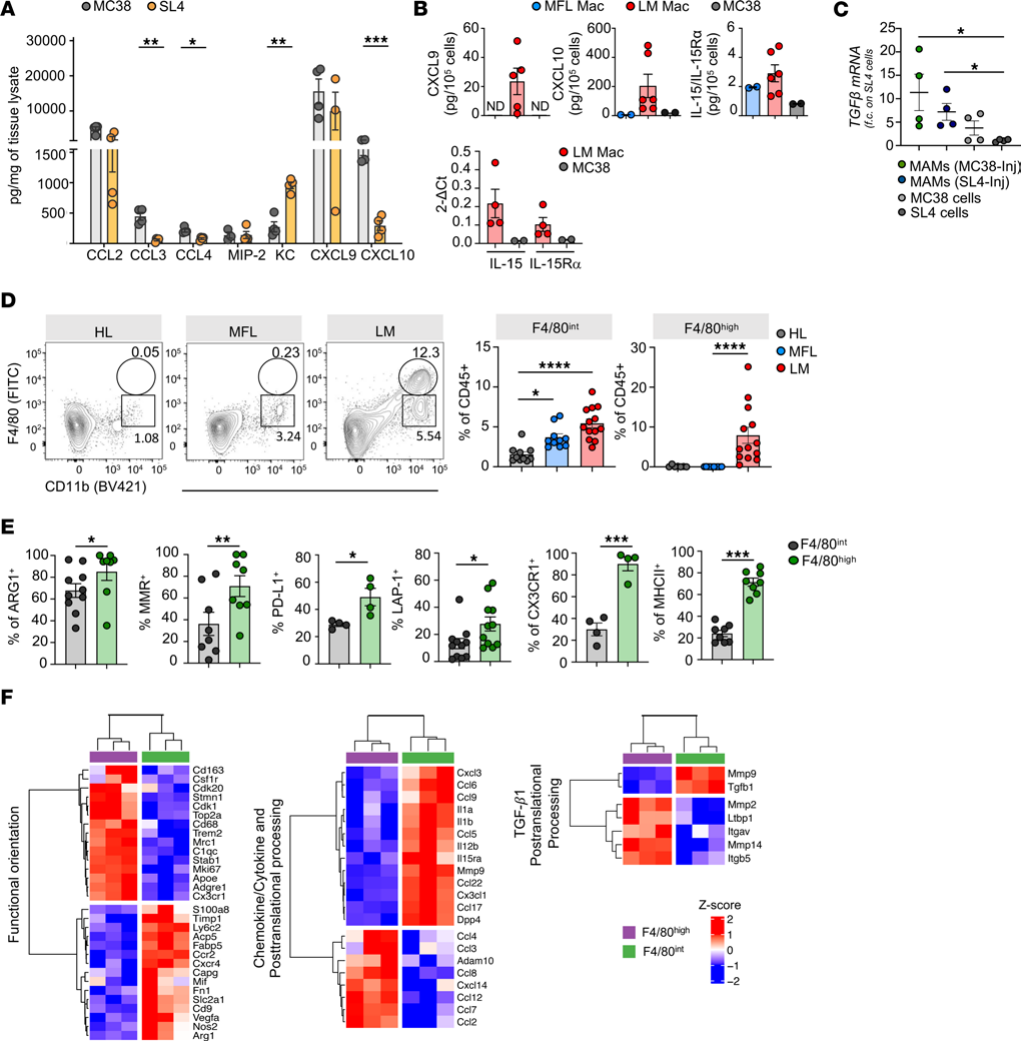
Macrophages showing TAM-like features accumulate in MC38-induced liver metastasis and produce cytokines and chemokines. (**A**) Histograms show chemokine concentrations in LM homogenates from MC38- and SL4-injected mice by luminex assay performed in duplicate (*n* = 3; pg/mg of tissue lysate) (CCL3 *P = 0.01, CCL4 *P = 0.04, KC *P = 0.01, ***P = 0.0007, 2-tailed Student’s *t* test). (**B**) Top, quantification of CXCL9, CXCL10, and IL-15/IL-15Ra complex secretion in MFL and LM macrophage- and MC38 cell-conditioned supernatants (*n* ≥ 2). Bottom, mRNA levels of *Il-15* and *Il-15R*α expression by macrophages isolated from MFL and LM and by MC38 cells (*n* = 2). (**C**) mRNA levels of TGF-β1 expression by MC38- and SL4-derived LM macrophages and MC38 and SL4 cell lines (*n* = 4, *P = 0.02, 2-tailed Student’s *t* test). (**D**) Contour plots show macrophage subsets HL and MC38-derived MFL and LM. Histograms represent mean frequency ± SEM of F4/80^int^ (CD11b^hi^F4/80^int^) and F4/80^hi^ cells (CD11b^hi^F4/80^hi^) gated on live lin^–^CD45^+^Ly6C^low/–^Gr1^–^ from at least 12 total mice. (*P = 0.02, ****P < 0.0001, 1-way ANOVA). (**E**) Histogram plots show expression of Arg1, MMR, PD-L1, LAP-1, CX_3_CR1, and MHCII in F4/80^int^ and F4/80^hi^ from MC38-induced LM (Arg1 *P = 0.017, MMR **P = 0.001, PD-L1 *P = 0.02, LAP-1 *P = 0.014, CX_3_CR1 ***P = 0.0005, MHCII ***P = 0.0001, 2-tailed Student’s *t* test) (**F**) Heatmaps showing DEGs from bulk RNA-seq of sorted F4/80^hi^ (violet) and F4/80^int^ (green) macrophages from MC38-derived LM.

IL-15 and TGF-β1 are crucial in directing type 1 ILC plasticity in several pathological contexts where CD49a^+^ NK cells have been found (8, 14). This suggests that NK cell phenotype and function in metastasis may depend on their ability to head toward tissue niches where IL-15 and TGF-β1 are coexpressed. Thus, we analyzed expression of chemotactic factors in MC38- and SL4-induced LM homogenates by luminex assay. We found expression of several chemokines that may contribute to type 1 ILC localization, including CXCL9, CXCL10, CCL2, CCL3 and CCL4 (Figure 5A). Furthermore, the expression levels of CCL3, CCL4, and CXCL10 were lower, whereas CXCL1/KC was higher in SL4 compared with MC38 metastases.

To identify the cellular source of IL-15, TGF-β1, and chemokines, we analyzed the myeloid compartment, which regulates NK cell phenotype and function in multiple pathological conditions, including CRC liver metastasis (9, 36). We found that macrophages and dendritic cells (DCs) accumulated both in MFL and LM in MC38 metastasis (Figure 5D and Supplemental Figure 6, A and B).

Metastasis-associated macrophages (MAMs) isolated from MC38-induced metastasis prominently expressed *Il-15* and *Il-15R*α transcripts and secreted CXCL9, CXCL10, and IL-15/IL-15Rα proteins at higher levels than MFL MAMs and MC38 cells (Figure 5B). Furthermore, although MAMs did not secrete TGF-β1, we could detect *TGF-*β*1* mRNA by RT-PCR (Figure 5C and Supplemental Figure 6C).

In MC38-induced MFL and LM, 2 main macrophage subsets could be distinguished based on the expression levels of the macrophage marker F4/80. Metastases were prevalently infiltrated by a unique CD11b^hi^F480^hi^ macrophage population endowed with enhanced expression of CD206, Arginase 1, TREM2, PD-L1, CX3CR1, CD115; variegated expression of class II MHC; and reduced Ly6C levels in comparison to the F4/80^int^ macrophage subset (Figure 5, D and E and Supplemental Figure 6, D and E). Moreover, CD11b^hi^F480^hi^ macrophages express higher levels of membrane latent associated protein (LAP1), indicating a more efficient TGF-β1 transport toward the plasma membrane (Figure 5E). In contrast, the tissue resident macrophage marker Tim4 was barely detectable on both cell populations (Supplemental Figure 7A), supporting the observation that liver MAMs originate from inflammatory monocytes and not from liver resident Kupffer cells (37).

To better characterize their molecular features, we performed bulk RNA-Seq on CD11b^hi^F4/80^int^ and CD11b^hi^F4/80^hi^ MAMs isolated from MC38-induced liver metastasis. DEG analysis shows that F4/80^int^ MAMs expressed high levels of transcripts identifying classical monocytes, including *Ly6c2*, *Ccr2*, *Sell*, and *S100a8,* which were strongly downregulated in F4/80^hi^ MAMs (Figure 5F). This suggests that F4/80^int^ MAMs represent recruited monocytes undergoing macrophage differentiation.

F4/80^hi^ MAMs expressed higher levels of proliferation-related genes (*Mki67, Stmn1, Cdk1,* and *Top2a*) and a different pattern of cytokine/chemokine genes compared with F4/80^int^ MAMs (Figure 5F). F4/80^hi^ MAMs also exhibited higher levels of transcripts for proteins involved in several aspects of TGF-β1 posttranslational regulation, which, along with higher expression levels of the LAP-1, suggest that TGF-β1 is preferentially activated on these cells (Figure 5F).

### F4/80^hi^ MAMs from MC38 but not SL4-induced metastasis include a MHCII^+^ subset expressing CXCL9 and TGF-β1.

**Figure 6:**
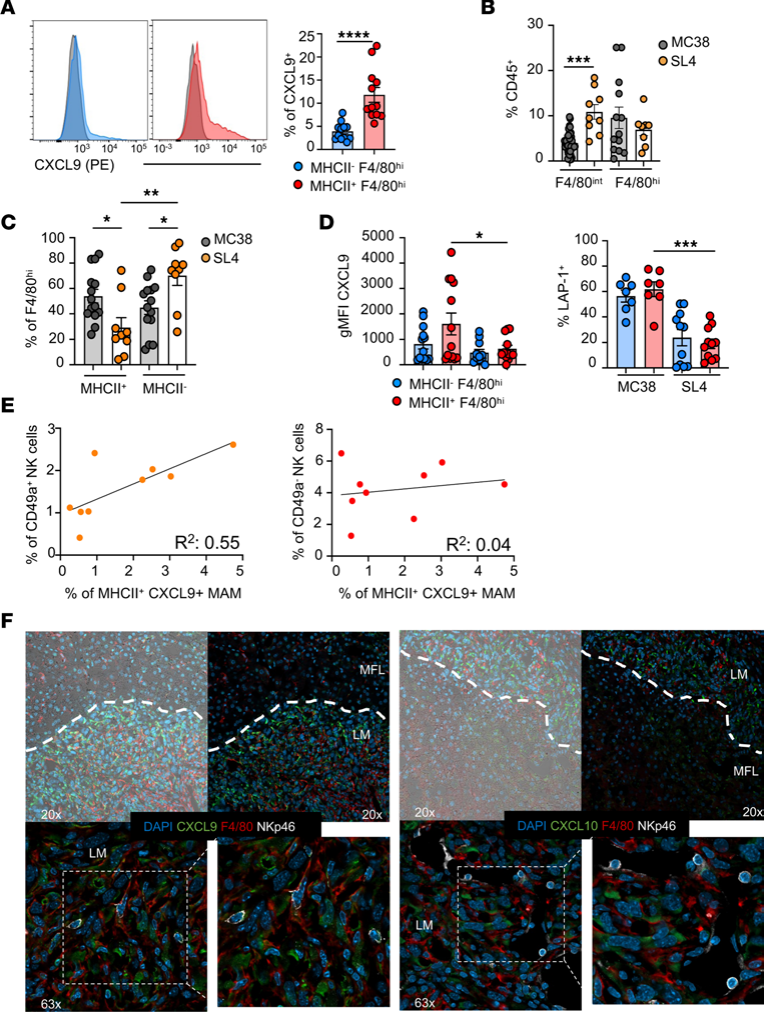
F4/80^hi^ MAMs from MC38 but not SL4-induced metastasis include subsets expressing CXCL9 and TGF-β1. (**A**) Representative FACS plots of CXCL9 expression on MHCII^+^ (red) or MHCII^–^ (blue) F4/80^hi^ macrophages in LM. Grey, isotype control (IC) staining. Histogram graph shows mean frequency ± SEM of CXCL9^+^ cells among MHCII^+^ or MHC-II^–^ F4/80^hi^ macrophages in MC38-injected mice (****P < 0.0001, 2-tailed Student’s *t* test). (**B**) Comparison of F4/80^int^ and F4/80^hi^ MAM mean frequency ± SEM between MC38-injected and SL4-injected mice (***P = 0.0002, 1-way ANOVA). (**C**) Mean frequency ± SEM of MHCII^+^ or MHCII^–^ cells among F4/80^hi^ MAMs in MC38- and SL4-induced LM (*n* > 9, *P = 0.04, **P = 0.001, 1-way ANOVA). (**D**) Comparison of CXCL9 gMFI and of frequency of LAP-1^+^ cells on MHCII^+^ or MHCII^–^F4/80^hi^ macrophages in MC38- or SL4-injected mice. Mean ± SEM of at least 7 mice per group in 2 independent experiments (gMFI *P = 0.029, LAP-1 ***P = 0.0003, 1-way ANOVA). (**E**) Correlation between frequency of CD49a^+^ or CD49a^–^ NK cells and matched MHCII^+^CXCL9^+^F4/80^hi^ populations among CD45^+^ cells in LM from MC38-injected mice. Simple linear regression of correlation and Pearson correlation coefficient (R^2^) are shown (CD49a^+^ NK *P* = 0.02, CD49a^–^ NK *P* = 0.62). (**F**) Immunofluorescence staining of liver metastasis: in blue DAPI staining for nuclei, in red F4/80, in green CXCL9/CXCL10 and in white NKp46. Top (original magnification, ×20), dotted line indicates tumor margin. Bottom, images at original magnification, ×63, with corresponding insets.

FACS analysis indicated that CXCL9 was prevalently expressed by F4/80^hi^ MAMs, compared with F4/80^int^ MAMs and DCs (Supplemental Figure 6F) and was mainly restricted to cells expressing class II MHC (F4/80^hi^MHCII^+^ macrophages) (Figure 6A), which represent an inflammatory macrophage population with a favorable predictive role in immunotherapy (38). Conversely, SL4-induced metastases were characterized by a higher expansion of F4/80^int^ and reduced MHCII^+^F4/80^hi^ macrophages expressing lower CXCL9 and LAP-1 levels when compared with the MC38 model (Figure 6, B–D).

We found a marked correlation between F4/80^hi^MHCII^+^CXCL9^+^ MAM and CD49a^+^ but not CD49a^–^ NK cell infiltration in MC38-induced metastasis, suggesting that CXCL9 expression by macrophages is causatively related to CD49a^+^ NK accumulation (Figure 6E). To understand whether NK cells localize in proximity to macrophages and/or CXCR3 ligands, we performed immunofluorescence staining of MC38-induced metastatic liver. CXCL9 and CXCL10 staining was detected in LM but not in MFL area. We found that all NK cells (NKp46^+^ cells) in LM localize together with macrophages in CXCL9 and CXCL10-rich areas (Figure 6F). A relevant fraction of macrophages stained positive for the chemokines, but, due to their diffuse expression, we cannot exclude that, in addition to macrophages, other cell types can secrete CXCL9/10.

Taken together, we show that the macrophage population expressing CXCL9 arises in immunogenic metastatic environment and colocalize with NK cells.

### Macrophages from MC38-induced metastasis contribute to CD49a^+^ NK cell generation.

**Figure 7:**
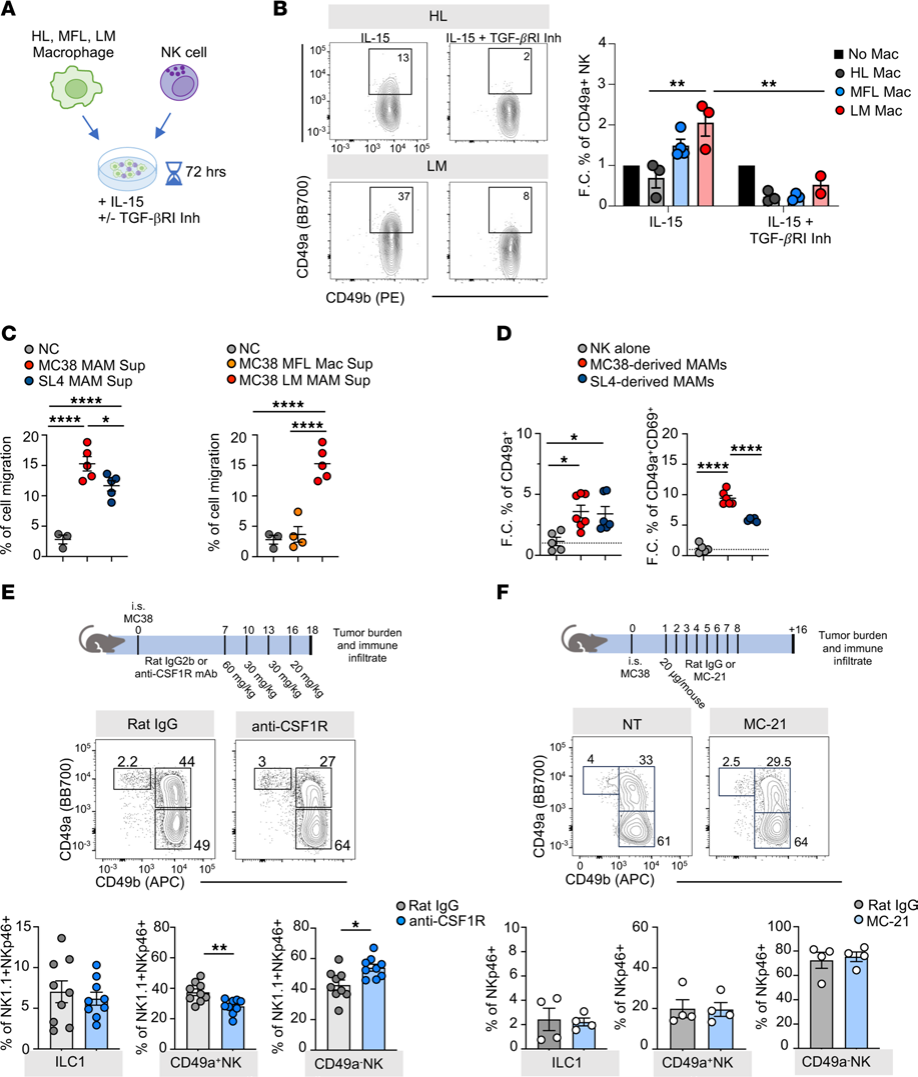
MAMs specifically induce acquisition of CD49a^+^ NK cell features by NK cells in a TGF-β1–mediated manner. (**A**) Experimental scheme of macrophage-NK cell coculture in vitro. (**B**) Lef,: representative contour plot of CD49a and CD49b expression by NK cells upon 72 hours coculture with macrophages purified from HL and LM with or without TGF-β-RI inhibitor (SB431542, 2.5 nM). Right, fold change (F.C.) of frequency ± SEM of CD49a^+^ cells among NK cells cultured with macrophages from HL, MFL, or LM relative to NK cells cultured alone (IL-15 *P = 0.03, IL-15+TGF-β-RI inhibitor *P = 0.044, 2-tailed Student’s *t* test in HL Mac versus LM Mac in IL-15 and LM Mac in IL-15 versus IL-15+ TGF-β-RI inhibitor). 3 experiments were performed in duplicate. (**C**) Left, percent of migration of splenic NK cells in response to supernatants collected from MC38- or SL4-derived LM macrophages and no chemokine control (NC) (n > 3, *P = 0.01, ****P < 0.0001, 1-way ANOVA). Right, percent of migration in response to MC38-derived MFL, LM macrophage supernatants and NC (*n* > 3, ****P < 0.0001, 1-way ANOVA). (**D**) Fold change of CD49a (left) and frequency of CD49a^+^CD69^+^ cells (right) in NK cells cultured alone or with LM-derived macrophages from MC38 and SL4 LM (*n* = 5, *P < 0.02, ****P < 0.0001, 1-way ANOVA). (**E** and **F**) Top, experimental workflow of in vivo treatment with anti-CSF1R and with MC-21. (**E**) Bottom, representative contour plots show ILC1, CD49a^+^ and CD49a^–^ NK cells in LM of control mAb (Rat IgG2a)-treated and anti-CSF1R-treated tumor-bearing mice. Numbers in plots indicate frequency among NK1.1^+^NKp46^+^ cells. Histogram plots show mean frequency ± SEM (*n* = 9 total mice in 2 independent experiments, CD49a^+^ NK **P = 0.003, CD49a^–^NK *P = 0.02, 2-tailed Student’s *t* test). (**F**) Bottom, representative contour plots show ILC1, CD49a^+^, and CD49a^–^NK cells in LM of control mAb (Rat IgG2a)-treated and MC-21–treated tumor-bearing mice. Numbers in plots indicate frequency among NK1.1^+^ NKp46^+^ cells. Histogram plots show mean frequency ± SEM (*n* = 4, 2 independent experiments were performed).

Hereafter, to understand whether MAMs are involved in NK cell phenotypic and functional shaping, we cultured NK cells alone or with macrophages isolated from HL, MFL, or the excised LM derived from MC38 injection (Figure 7A). To sustain NK cell survival, all conditions were supplemented with low levels of IL-15 (10 ng/mL). NK cells cocultured with MAMs prominently upregulated CD49a compared with NK cells in the other conditions and this effect was inhibited by pharmacological blockade of TGF-β1 receptor signaling (Figure 7B). The effect was markedly reduced when cell-to-cell contact was abrogated by using transwells, demonstrating a relevant role of NK cell/macrophage interaction in CD49a^+^ NK cells generation (Supplemental Figure 7B). Although less pronounced, an induction of CD49a was also observed through NK cell coculture with MFL macrophages, suggesting that macrophages in the inflamed liver also have the potential to induce CD49a^+^ NK cells in vivo. However, when purified NK cells were exposed to supernatants obtained from MFL versus LM macrophages in cell migration assays, only MAM supernatant promoted NK cell chemotaxis, indicating that MFL macrophages may be incapable of attracting and interacting with NK cells in the tissue environment (Figure 7C). CD49a^+^CD49b^+^ NK cells generated upon coculture with MAMs, or by in vitro culture in media supplemented with a combination of IL-15 and TGF-β1, exhibited higher degranulation capacity than NK cells cultured alone (Supplemental Figure 7C).

A comparable induction of CD49a was observed when NK cells were cocultured with SL4-induced MAMs, but NK cells showed reduced chemotaxis to SL4-derived MAM supernatants and a significant decrease in proliferation capacity and expression of the residency marker CD69, compared with coculture with MC38-induced MAMs (Figure 7D and Supplemental Figure 7D). Although tumor cells produce TGF-β1, we did not observe upregulation of CD49a expression on NK cells upon coculture with MC38 cells or incubation with MC38-derived supernatant (data not shown).

Altogether, these results suggest that MAMs contribute to the generation of tissue niches critical for NK cell transdifferentiation into CD49a^+^ NK cells by presenting CXCR3 ligand and TGF-β1. These observations in mouse models prompted us to investigate the cellular interactions between the human CXCR3_NK cluster and different macrophage clusters identified in single-cell RNA-seq (scRNA-seq) dataset of MFL and LM. The analysis showed that metastatic Macro_C1QC and CXCR3_NK were enriched in CXCL10-CXCR3 interactions. Other ligand-receptor interactions, including IL15-IL2RG, were differently enriched in macrophage clusters and in MFL versus LM, strengthening the translation relevance of CXCR3-mediated NK cell–macrophage interplay (Supplemental Figure 7E).

We next addressed whether MAMs were involved in CD49a^+^NK cell differentiation in vivo. In vivo administration of anti-CSF1R blocking mAb 7 days postinjection led to almost complete (greater than 90%) depletion of LM F4/80^hi^ macrophages with minimal effects on F4/80^int^ macrophages and metastasis burden (Supplemental Figure 8, A–C). These experiments showed reduced frequency of CD49a^+^CD49b^+^ cells in metastasis of mice treated with anti-CSF1R mAb compared with control mAb-treated mice, which corresponded to a marked increase of CD49a^–^ NK cell infiltration and to reduced CXCR3 ligand expression in total LM and in MAM left overs upon (Figure 7E and Supplemental Figure 8D). The treatment did not affect frequency of bone marrow monocytes and dendritic cells (Supplemental Figure 8E).

Since CD49a upregulation was only partially inhibited by F4/80^hi^ MAMs, other macrophage populations could participate or compensate for F4/80^hi^ MAM depletion to promote CD49a^+^ NK cell generation. LM MAMs derive from monocytes, whose recruitment critically depends on the chemokine receptor CCR2 (37). Thus, we depleted inflammatory monocytes by in vivo administration of an anti-CCR2 monoclonal antibody (clone MC-21, (39)) and analyzed the distribution of macrophages and NK cells within metastasis. Anti-CCR2–mediated depletion of monocytes led to a marked decrease of F4/80^int^ MAMs (greater than 65%) but only a mild decrease of F480^hi^ MAMs (less than 35%), which can be explained by MAM self renewal and replenishment upon monocyte depletion (37) (Supplemental Figure 8F). Anti-CCR2 treatment demonstrated no relevant effect on metastasis burden nor on CD49a^+^ NK cell accumulation, suggesting that F4/80^int^ MAMs have a negligible role in NK cell plasticity (Figure 7F and Supplemental Figure 8F). Overall, we demonstrated that, among LM macrophages, F4/80^hi^ MAMs contribute to NK cell plasticity through TGF-β1 production.

### CXCR3 receptor/ligand axis controls CD49a^+^NK cell generation in metastatic nodules.

We have shown that MAMs support the development of NK cell phenotypes associated with metastasis and produce CXCR3 ligands within metastatic nodules, which regulate NK cell LM infiltration.

CXCR3 promotes tumor infiltration of several immune cells that can play redundant or antagonistic roles in the TME, leading to conflicting results on the function of CXCR3/ligand axis in tumor progression. Thus, we generated Ncr1^greenCre^*Cxcr3*^fl/fl^ mice (*Ncr1*^ΔCxcr3^) that lack *Cxcr3* specifically in NKp46^+^ cells. CXCR3 was efficiently ablated from NKp46^+^ cells but not from CD3^+^ cells, as assessed by flow cytometry analysis of liver lymphocytes (Supplemental Figure 9A).

The *Ncr1* conditional deletion of *Cxcr3* had no effect on the homeostatic pool of NKp46^+^ cells in liver or in other organs (Supplemental Figure 9B and data not shown), consistent with no alteration observed in mice carrying germline deletion of *Cxcr3* (40). Moreover, we did not observe any effect on other liver immune cell type tissue distribution under steady state (Supplemental Figure 9B).

**Figure 8:**
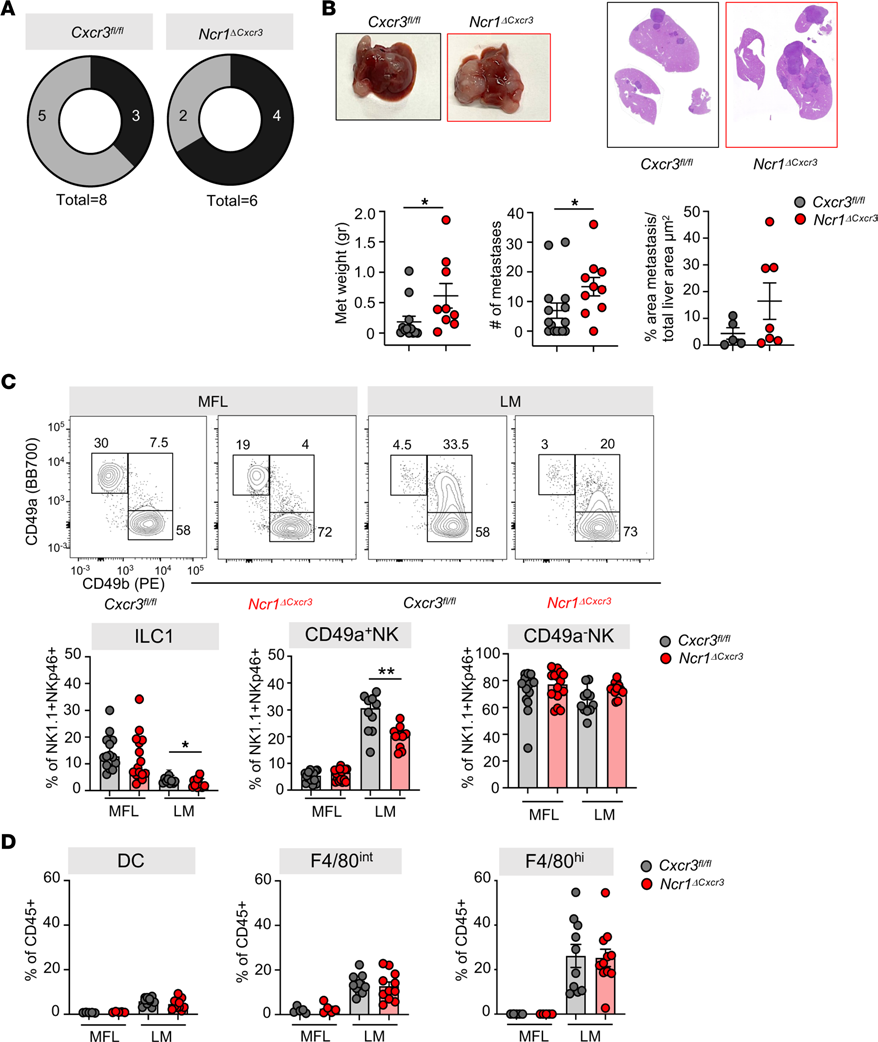
Deletion of *Cxcr3* in *Ncr1^+^* ILCs impairs CD49a^+^ NK generation and accelerates metastasis formation. (**A**) Cake plots show incidence of LM in Cxcr3^fl/fl^ and Ncr1^ΔCxcr3^ mice 15 days p.i. 2 independent experiments were performed. (**B**) Top, representative pictures of metastatic livers and hematoxylin-eosin staining. Bottom, scatter plots show mean ± SEM of metastasis weight (gr) and number and quantification of liver section from Cxcr3^fl/fl^ (grey) and Ncr1^ΔCxcr3^ (red) mice 20 days p.i. 3 independent experiments were performed (Met Weight *P = 0.012, no. metastases *P = 0.022, 2-tailed Student’s *t* test). (**C**) Representative contour plots of ILC1, CD49a^+^ NK and CD49a^–^ NK cells in MFL and LM from Cxcr3^fl/fl^ and Ncr1^ΔCxcr3^ mice. Numbers in plots correspond to frequency. Histograms show corresponding median frequency with 95% CI among NK1.1^+^ NKp46^+^ cells (ILC1 *P = 0.026, CD49a^+^ NK **P = 0.0015, 2-tailed Student’s *t* test to compare Cxcr3^fl/fl^ and Ncr1^ΔCxcr3^ cell frequency). (**D**) Histogram plots show frequency of DCs, F4/80^int^ and F4/80^hi^ macrophages in MFL and LM of Cxcr3^fl/fl^ and Ncr1^ΔCxcr3^ tumor-bearing mice among CD45^+^ cells. Data are presented as mean ± SEM. Three independent experiments were performed.

To evaluate the cell-intrinsic role of CXCR3 in type 1 ILC hepatic and metastasis infiltration and metastasis formation, we next induced liver metastasis in *Cxcr3^fl/fl^* and *Ncr1*^ΔCxcr3^ mice by MC38 intrasplenic injection. We chose days 15 and 20 postinjection as endpoints to assess differences at intermediate and advanced stages of tumor growth, respectively. Tumor burden and metastasis incidence in control *Cxcr3^fl/fl^* mice were reduced at 15 days compared with 20 days (data not shown). Interestingly, *Ncr1*^ΔCxcr3^ mice displayed increased metastasis incidence at 15 days and increased tumor burden at 20 days postinjection, indicating a role for CXCR3^+^NKp46^+^ cells in protection from metastasis (Figure 8, A and B). CD49a^+^ NK cells infiltrating the metastatic nodules in *Cxcr3^fl/fl^* mice were significantly reduced in *Ncr1*^ΔCxcr3^ mice, which corresponded to a mild increase of CD49a^–^ NK cells (Figure 8C). Moreover, we did not observe differences in LM macrophage or DC frequency in *Cxcr3^fl/fl^* and *Ncr1*^ΔCxcr3^ mice, indicating that CXCR3 regulates CD49a^+^ NK cell accumulation by promoting NK cell recruitment and in situ NK cell activation by macrophages rather than by modifying other immune cell infiltration (Figure 8D).

Altogether, these results unravel a role for the CXCR3/ligand axis in directing NK cell positioning within the metastatic nodules and may offer new insight into the mechanisms governing immune cell crosstalk in TME that participate to the restraining of metastasis growth.

## Discussion

The mortality of CRC remains significantly impacted by liver metastasis (41), with the liver consistently emerging as a preferred metastatic site for various solid tumors. This highlights the unique immunological attributes of the hepatic environment (42). Type 1 ILCs constitute a significant proportion of innate lymphocytes in the liver, with both circulating cNK cells and trILC1s playing a substantial role in liver immune surveillance against metastasis formation (8, 43).

By examining the transcriptional programs activated in type-1 innate lymphocytes and investigating the role of chemokine receptors in experimental mouse models of CRC liver metastasis, we deciphered the cell-state transitions that underlie NK cell reshaping by the metastasis environment. Here, we demonstrated that during NK cell response to MC38-induced metastasis, NK cells adopt at least 2 divergent transcriptional states with CD49a^+^ cells progressively acquiring ILC1 features and CD49a^–^ cells remaining phenotypically similar to MFL NK cells. These changes are characterized by the acquisition of a tissue-residence phenotype and enhanced effector functions in CD49a^+^ ILC1-like NK cells that correlate with efficient metastasis infiltration and persistence and improved cytotoxic capacity. This functional adaptation may reflect acquisition of ILC1 features allowing counteraction of tumor growth, as liver ILC1s, which include potent cytotoxic cells able to kill tumor cells, are virtually excluded from metastatic lesion (7, 8). Mechanisms responsible for low ILC1 metastasis infiltration may rely on intrinsic expression of retention markers (i.e., CXCR6), which do not allow efficient translocation from liver to tumor and will require further investigation.

Our findings reveal that metastasis infiltration by NK cells relies at least in part on CXCR3-mediated migration, and that preventing CXCR3-mediated tissue migration of NKp46^+^ cells through genetic deletion is sufficient to hinder the acquisition of an ILC1-like cell phenotype by NK cells. We hypothesize that CXCR3^+^ NK cells can promptly infiltrate the metastasis and are preferentially exposed to a F480^hi^MHCII^+^ macrophage subset able to promote their transdifferentiation into ILC1-like cells thanks to local recruitment supported by macrophage-produced CXCR3Ls.

By demonstrating that MAMs can produce IL-15 and TGF-β1 in the same niche, our data expand previous observations showing that the combined action of these cytokines promotes the acquisition of an activated CD49a^+^ phenotype by NK cells (14, 44). Although our finding is supported by recent evidence of preserved or even increased NK cell function in the presence of TGF-β1 (45), this cytokine is a well-established inhibitor of NK cell activation and function. A possible explanation for TGF-β1–positive effect on functional ILC1-like NK cell differentiation may stand on its ability to induce molecules involved in immune cell tissue residency, which may support NK functionality by allowing access to IL-15–rich niches (45–47). Indeed, we observed that the preferential expression of CD49a and CD69 correlated with the ability of NK cells to infiltrate the tumor via CXCR3. In contrast, LM CXCR3^–^ NK cell subsets do not express CD49a and CD69 and have reduced cytotoxic function, possibly due to their exposure to environmental signals distinct from those provided by CXCL9-expressing macrophages. Indeed, these effects are partially replicated when *Cxcr3* expression in NKp46^+^ cells is missing due to genetic deletion. Consistently, a similar reduction of CD49a^+^ ILC1-like cell metastasis infiltration was observed in SL4-induced metastasis, characterized by low frequency of CXCL9^+^F4/80^hi^MHCII^+^ macrophages. Nevertheless, the modest accumulation of CD49a^+^ NK cells observed in SL4-induced metastasis in WT mice and MC38-induced metastasis in *Cxcr3-*deficient mice suggests that, in addition to CXCR3, other chemokine receptors participate in NK cell recruitment and plasticity in LM.

Our results diverge from recent findings that clearly demonstrates a rapid loss of effector functions by NK cells entering MC38 tumors growing subcutaneously, corresponding to acquisition of CD49a and loss of the maturation marker CD11b (28). Nevertheless, we observed only a mild reduction of CD11b expression by CD49a^+^ NK cells infiltrating metastasis, indicating that the unique liver immune features affect the MME differently from other anatomical sites.

A prometastatic role of macrophages in the liver has been ascertained in several studies, including mouse CRC models (48–50), while macrophages were also shown to enhance NK cell function by various means in the MC38 model (9, 10). We found that CD49a^+^ NK cell accumulation correlated with CXCL9^+^MHCII^+^ MAM, showing features of an inflammatory macrophage population recently identified in mouse and human tumors and linked to better clinical outcome (32, 38, 51–53). Correspondingly, we found enrichment of CXCR3^+^ NK clusters with residency features in human CRC liver metastasis samples compared with metastasis-free liver or PB.

CXCL9 is documented as a favorable prognostic marker in several cancers, including CRC (54). This observation suggests a potential role of F4/80^hi^MHCII^+^CXCL9^+^ MAM in fostering a proinflammatory and antitumoral niche within the metastatic site. Accumulation of this MAM population in MC38 and not in SL4 correlates with the preferential accumulation of CD49a^+^ NK cells and might better counterbalance the activity of protumoral MAMs in the former model. Indeed, we showed that F4/80^hi^ MAM depletion negatively impacted on CD49a^+^ NK cell frequency in MC38-induced metastasis, supporting the hypothesis that macrophage-NK crosstalk is important in shaping NK cell phenotype in this context. One limitation of this study is the requirement for low concentration of exogenous IL-15 in NK cell–MAM coculture experiments, which is essential to maintain NK cell viability but may have diminished differences in the effects of MFL versus LM, as well as MC38- versus SL4-derived macrophages. Nonetheless, we were still able to appreciate differences in NK cell residency, proliferation features, and chemotactic behavior promoted by macrophages obtained from the different metastatic environments.

We also showed that CXCR3^+^ NK cell signature in LM predicts better prognosis in patients with CRC. Although the mechanism of NK_CXCR3 activation is still unknown, by analyzing the ligand-receptor pair communication, we found that the LM-enriched Macro_C1QC subset could interact with NK_CXCR3 cells through CXCL10-CXCR3, but the prognostic role of this interaction will require further investigation with a larger population cohort.

CXCR3 has been extensively characterized as an antitumoral chemokine receptor, influencing the infiltration of NK and T cells in various primitive tumors. Here, we show for what we believe to be the first time that CXCR3 helps to preserve NK cell functionality and antimetastatic function by facilitating their conversion into CD49a^+^ NK cells with ILC1-like features. Preventing CXCR3-mediated NKp46^+^ cell positioning within tissue by *Cxcr3* genetic deletion was sufficient to impair CD49a^+^ NK cell accumulation in metastasis and correlated with enhanced metastasis burden.

Collectively, these findings suggest a role played by the CXCR3 receptor/ligand axis in orchestrating NK cell infiltration and localization within specific metastatic niches. Thus, regulation of NK cell metastasis infiltration by potentiating CXCR3 responsiveness may represent a valid therapeutic strategy to dictate the outcome of the anticancer immune response.

## Methods

### Sex as a biological variable.

Our study analyzed male and female animals, and sex was not considered as a biological variable.

### Animals.

C57BL/6J, CD45.1 (Jackson mouse stock no. 008449) and *Cxcr3^–/–^* genetically knocked-out female mice were purchased from Charles River. *Ncr1^greenCre/+^*mice (55) were crossed with *Cxcr3^fl/fl^* mice (56) to obtain *Ncr1^greenCre/+^*;*Cxcr3^fl/fl^* (*Ncr1*^ΔCxcr3^) mice. For experiments in *Ncr1*^ΔCxcr3^ mice, both males and females were used, and littermates were used as controls. Experimental groups were randomized according to sex and age. Animals were maintained in the animal facility of the Department of Anatomical, Histological, Medical Legal Science and Locomotor Apparatus of Sapienza University of Rome under specific pathogen-free conditions in accordance with the guidelines of Animal Care and Use Committee of the Health Minister. Mice were kept in rooms with a 12-hour light/dark cycle and 22°C temperature, housed in environmental enriched cages and were fed ad libitum. All efforts were made to minimize the number of animals used and their suffering.

### Cancer cell lines.

In vivo experiments were conducted using the murine MC38 (originally obtained in house from ATCC (57)) or SL4 adenocarcinoma cell line syngeneic with C57BL/6 mice, provided by Tatsuro Irimura (University of Tokyo, Tokyo, Japan). MC38 and SL4 cells were cultured under standard conditions in complete RPMI-1640 or DMEM (1% nonessential amino acids, 1% sodium pyruvate [Gibco]), respectively, supplemented with 10% heat-inactivated FBS, 2mmol/L glutamine, and antibiotics (100IU/mL penicillin). Cell lines were periodically authenticated by morphologic inspection, verified to be mycoplasma free, and were passaged for no more than 4 weeks from thawing. For intrasplenic injection, MC38 or SL4 cells were detached using EDTA 1mM, filtered through a 70μm cell strainer (Corning) and resuspended in PBS (EuroClone) 0.01% EDTA.

### Antibodies and other materials.

Anti-mouse antibodies against the following antigens for flow cytometry (clone in parentheses) were used: NK1.1-BV510/BV650/APC (PK136), CD16/32 (clone 93), Arg1-APC (A1EXF5), CD3-BV605/FITC (145-2C11), CD8a-FITC (53.6-7), CD11b-PE/PE-Cy7/FITC/BV510/BV421/APC-R700 (M1/70), CD11c-BV711/PE-Cy7 (N418), CD19-BV605/FITC (1D3), CD27-FITC (LG.7F9), CD39-PE-Cy7 (Duha59), CD45.1-PE-Cy7 (A20), CD45.2-PE/APC-H7/BUV395 (clone 104), CD49a-BB700 (Ha31/8), CD49b-APC/BV421/PE (DX5), CD69-AF488/PECF594 (H1.2F3), CD103-PerCP M290), CD73-BV421 (TY/11.8), CD62L-PE (MEL-14), CXCR6-PECy7 (SA051D1), CXCR3-APC/BV421 CXCR3-173), CX3CR1-PE (SA011F11), CXCR4-PE (2B11), EOMES-PE-Cy7 (Dan11Mag), F480-FITC/BV421 (BM8), Gr1-PerCP (RB6-8C5), iNOS-PE (CXNFT), KLRG1-BV786 (2F1), Ly6C-BV510/APC/BV605 (HK1.4), Ly6G-PE (1A8), MMR-PE-Cy7 (C068C2), PD-L1-PE/BV785 (MIH5), LAP-1-FITC/PE (TW7-16B4), MHCII-APC (M5/114.15.2), TREM2 APC, NKp46-BV421/PE/PE-Dazzle594 (29A1.4), NK1.1-BV650 (PK136), TIGIT-PE (1G9), Ki-67-FITC (SOLA-15), Perforin-PE (S16009A), CD107a-FITC/APC (1D4B), IFN-γ-APC/BV421 (XMG1.2), TNF-a-PE-Cy7 (MP6-XT22), CXCL9 (MIG-2F5.5), Tim4-AF647 (RMT4-54), CD115-PE (AF598) anti-bromo-deossi-uridina APC (bu20A). Antibodies were purchased from R&D Systems, Thermo Fisher Scientific, and Biolegend.

For microscopy, the following antibodies were used: NKp46/NCR1 (Polyclonal Goat IgG AF2225, R&D), anti-human/mouse CXCL9 PE (11H1L14, Thermo Fisher Scientific), F4/80 (clone A3-1, Biorad), CXCL10 (10H11L3, Invitrogen).

For in vivo experiments, the following antibodies were used: Avertin (Sigma), Zoletil, Rompum (Demas s.r.l.) and Metacam (Meloxicam) (Boehringer Ingelheim), Betadine 10% (Bayer). In vitro experiments and flow cytometry: PBS, PBS with Ca^2+^ and Mg^2+^ (EuroClone), paraformaldehyde (PFA), RPMI-1640, Physiological solution, Red Blood Cell Lysis Buffer (Sigma-Aldrich), Liberase TL (Roche), DNase I (Roche), Percoll Density Gradient Media (GE Healthcare), Cytofix/Cytoperm TM Fixation/Permeabilization Kit was from BD Biosciences

### Model of experimental liver metastasis.

Liver metastases were induced in 8-10 week-old mice by injecting 3 × 10^5^ MC38 or SL4 cells in 60 μL PBS into the splenic parenchyma. Spleen was excised 5 minutes after injection to prevent splenic tumor growth. Peritoneum was sutured, and skin closed with autoclips. Control mice underwent mock surgery. Metacam was administered for 3 days for pain relief. Livers were analyzed 16 or 20 days postsurgery for SL4 and MC38, respectively. Total liver weight was registered, macroscopically visible metastases were counted, and, once excised, pooled and weighed alone.

### Depletion of cells by antibodies or drugs.

To deplete F4/80^hi^ MAM tumor-bearing mice were treated i.p. with anti-CSF1R (CD115, clone AFS98, BioXcell) or control Rat IgG2a (clone 2A3, BioXcell) mAbs as following: 60 mg/kg at day +7 intrasplenic injection, 30 mg/kg at days +10 and +13, and 20 mg/kg at day +16.

To deplete F4/80^int^ MAM tumor-bearing mice were treated i.p. with MC-21 mAb or Rat IgG control MC-67 mAb (provided by Matthias Mack, Universität Regensburg, Regensburg, Germany) as following: 20 μg/mouse everyday from day +1 to day +10 of intrasplenic injection.

### Cell isolation from tissue and flow cytometry.

Liver parenchyma was smashed, filtered (100 μm strainer, Corning), and washed twice with PBS. Liver metastases were shredded, digested in RPMI-1640 with 0.25 mg/mL Liberase TL and 0.5 mg/mL DNase I for 30 minutes at 37°C, and filtered (100 μm strainer). Leukocytes were enriched via 40% Percoll density gradient centrifugation. Blood was collected from the tail vein in heparin tubes, lysed with Red Blood Cell Lysis Buffer. Single cells were incubated with anti-CD16/32 for Fc blocking, stained for surface markers in 50 μL antibody mix, and washed with staining buffer (0.5% BSA, 2 mM EDTA, 0.025% NaN3). For intracellular staining cells were fixed (1 hour, RT, IC Fixation Buffer, eBioscience) and stained for intracellular markers (2 hours, 4°C). Dead cells were excluded using Live/Dead Fixable staining. Samples were acquired on a FACS Canto II or LSR Fortessa (BD Biosciences) and analyzed with FlowJo v10 (Tree Star).

For cell sorting, single-cell suspensions were stained with the indicated Abs and sorted using a FACSAriaIII (BD Biosciences) with 488 nm, 561 nm, and 633 nm lasers and FACSDiva v6.1.3. Following the gating strategy (Supplemental Figure 1A), cells were gated by FSC-A/SSC-A, doublets excluded via FSC (H versus A), and sorted as NK cells (live lin-CD45^+^NKp46^+^CD49a^–^CD49b^+^), ILC1 (live lin-CD45^+^NKp46^+^CD49a^+^CD49b^–^), and CD49a^+^ NK cells (live lin-CD45^+^NKp46^+^CD49a^+^CD49b^+^). Macrophages were sorted after CD45^+^ enrichment as CD45^+^CD11b^+^F4/80^low^ and CD45^+^CD11b^+^F4/80^hi^.

To minimize stress, cells were sorted using a 100 mm ceramic nozzle, 19.84 psi sheath pressure, 18.96 psi sample pressure, and less than or equal to 3,000 events/second acquisition rate. Postsorting, an aliquot was assessed for purity (greater than 95%).

For macrophage purification, liver parenchyma was digested with a Liver Dissociation Kit (Miltenyi Biotech), centrifuged (200 g, 15 minutes), and filtered (40 μm strainer). F4/80^+^ cells were purified using mouse anti-F4/80 MicroBeads Ultrapure (Miltenyi Biotech).

### Ex vivo assays.

For intracellular cytokine detection, cells were stimulated with 100 ng/mL recombinant mouse IL-12p70 (Peprotech) and 50 ng/mL IL-15 (R&D) for 4 hours at 37°C in complete RPMI-1640 (55 mol/L 2-ME) with brefeldin A. Cells were then stained for surface markers, fixed, permeabilized (Cytofix/Cytoperm kit, BD Biosciences), and stained for intracellular cytokines. For CD107a detection, cells were freshly stained with anti-CD107a mAb.

For the killing assay, cells from 6–8 pooled MFL or LM samples were sorted via FACS Aria III (BD Biosciences). Target MC38 cells were stained with 2.5 μM CFSE and incubated in complete RPMI-1640 for 6 hours at 37°C with effector cells. CFSE-labeled cells alone served as controls. Specific killing was calculated as: ([% cell death in the presence of NK cells] – [% spontaneous cell death]) / (100 – [% spontaneous cell death]).

### Adoptive transfer experiments.

NK cells were purified from spleens of *Cxcr3^+/+^Ly5.1* and *Cxcr3^–/–^Ly5.2* mice using NK cell purification kit (Miltenyi Biotech) and were pooled at 1:1 ratio and CFSE labeled. For competitive adoptive transfer, each recipient mouse received 1 × 10^6^ pooled CFSE^+^ NK cells by i.v. injection. Donor type 1 ILCs in the liver of recipient mice were identified as CD3^–^NK1.1^+^ cells coexpressing CSFE with CD45.1 or CD45.2 by flow cytometry 18 hours after transfer.

### In vitro NK cell culture and degranulation assay.

For NK cell in vitro culture, freshly purified splenic NK cells (50,000/well) were plated in U-bottom 96-well plates with complete RPMI-1640 (1% nonessential amino acids, 1% sodium pyruvate, 55 mol/L 2-ME) and 10 ng/mL IL-15 (R&D Systems), with or without 10 ng/mL TGF-β1 (Peprotech), at 37°C, 5% CO_2_ for 3 or 7 days.

For NK cell–macrophage coculture, 5 × 10^5^ macrophages (HL, MFL, or LM) purified with anti-mouse F4/80 + Microbeads UltraPure (Miltenyi Biotech) were plated in flat-bottom 96-well plates with complete DMEM for 3 hours, washed with PBS, and cocultured with splenic NK cells (1:1 ratio) in IL-15–supplemented medium for 72 hours. To reduce TGF-β1 levels, some experiments used 2% FBS RPMI-1640.

For CD107a detection, flat-bottom high-binding 96-well plates were coated overnight (4°C) with 5 μg anti-NKR-P1C (BioXCells). NK cells were incubated in complete medium with GolgiStop (BD Bioscience) for 4 hours, adding fluorochrome-conjugated CD107a antibody in the last 2 hours.

For NK-MC38 coculture, MC38 cells (live or apoptotic via heat shock: 5 minutes at 65°C, 5 minutes on ice) were incubated with NK cells at 1:1 or 1:3 ratios.

### In vitro migration assay.

After 2 hours’ recovery in complete medium (37°C), purified NK cells were loaded onto 96-well 5.0 μm transwell plates (Corning, 3388) with membranes preactivated with 1 μg/mL ICAM for 1 hour (37°C). NK cells migrated for 2 hours toward macrophage supernatants or control chemokine. Migration medium alone served as a negative control. Migration (%) was calculated as: (migrated cells / total loaded cells) × 100.

### BrdU Assay.

For the in vivo BrdU assay, mice received 1mg BrdU (i.p.) in PBS or DMSO 18 hours and 9 hours before sacrifice. After organ extraction, cells were stained.

For the in vitro BrdU assay, 48 hours after initiating MAM-NK coculture, cells were stimulated with 25 ng/mL IL-15. During the last 6 hours, 20 μM BrdU was added. After 72 hours, NK cells were harvested, stained for extracellular markers, washed (PBS, 0.5% BSA, 2 mM EDTA), and fixed (PBS, 30% methanol, 0.4% PFA) for 30 min (room temperature [RT]). Pellets were stored overnight at 4°C. The next day, cells were resuspended in 500 μL Ca^++^/Mg^++^-PBS with 250 μg/mL DNase for 15 minutes (RT), washed, stained with anti-BrdU APC or control IgG (1 hour, RT), washed again, and analyzed by flow cytometry.

### Quantitative Real-Time PCR.

Macrophages from liver metastasis were purified with UltraPure F4/80 Beads (Miltenyi Biotech) with at least 96% purity, seeded in flat bottom 96-well plate in complete DMEM for 3 hours, washed with PBS, and total RNA was extracted using Total RNA Mini kit (Geneaid Biotech), according to manufacturer’s instructions. Reverse transcription was carried out in a 25 μL reaction volume with 1μg of total RNA according to the manufacturer’s protocol for M-MLV reverse transcriptase (Promega). cDNAs were amplified in duplicate with primers for TGF-β1 (forward: CCTGTCCAAACTAAGGC, reverse: GGTTTTCTCATAGATGGCG) and GAPDH (forward: TCGTCC CGTAGACAAAATGG, reverse: TTGAGGTCAATGAAGGGGTC) IL-15 (Thermo Fisher Scientific, Mm00434210_m1) and IL-15Rα (Thermo Fisher Scientific, Mm04336046_m1). RNA expression level was measured using the comparative Ct (threshold cycle) method. All PCR reactions were performed using an ABI Prism 7900 Sequence Detection system (Applied Biosystems).

### Luminex Assay.

ILC1, CD49a^+^ NK and CD49a^–^ NK cells from MFL, LM, and spleens of healthy mice were cultured in RPMI-1640, while liver metastasis macrophages were cultured in DMEM 1% BSA. Supernatants were collected after 24 hours, spun twice to remove debris, and stored at –80°C. Tissues were homogenized using gentleMACS dissociator. Luminex assay (Thermo Fisher Scientific) was performed per manufacturer instructions, and samples were acquired with Bio-Plex MAGPIX.

### Histology.

Histology was performed on 4 μm FFPE liver sections, stained with H&E, and scanned using the PANNORAMIC DESK II DW scanner (3DHISTECH). Liver metastases were measured by tracing lesion perimeters in SlideViewer (3DHISTECH).

### IF and confocal microscopy.

Mice were overdosed with halothane and perfused intracardially with PBS and 4% PFA. After fixation, the liver was preserved in 4% PFA overnight, incubated in sucrose for 24–48 hours, and frozen at –80°C. Cryostat sections (20 μm) were washed in PBS, blocked (3% goat serum, 0.3% Triton X-100, 1 hour, RT), and incubated overnight (4°C) with primary antibodies in PBS (1% goat serum, 0.1% Triton X-100), including anti-NKp46 (1:50), anti-CD31 (1:200). After washes, sections were stained with fluorophore-conjugated secondary antibodies, Hoechst for nuclei, and analyzed by fluorescence microscopy. Images were digitized using a CoolSNAP camera (Photometrics) on an ECLIPSE Ti-S microscope (Nikon) and processed with MetaMorph 7.6.5.0 (Molecular Devices).

For immunofluorescence, 8 μm liver cryosections were washed in PBS, blocked (3% BSA, 30 minutes, RT), and incubated (1 hour, RT) with primary antibodies, including anti-NKp46 (1:50), anti-F4/80 (1:100), anti-CXCL9, or anti-CXCL10 (1:100) in PBS (0.5% BSA). After washes, sections were stained with fluorophore-conjugated secondary antibodies and DAPI for nuclei. Images were acquired using a Zeiss LSM980 confocal microscope.

### RNA-seq analysis.

Type-1 lymphocytes from 6–8 pooled MFL or LM samples were sorted for bulk RNA-seq. RNA was extracted using the Norgen Single Cell RNA Purification Kit, and quality was assessed via Nanodrop. Sequencing was performed by DNALINK Inc. on the Illumina Novaseq 6000 platform using the SMARTer Stranded Total RNA-Seq kit v2-Pico Input, generating stranded, paired-end 101 bp reads. Total yield ranged from 5.9–8.6 billion bases, with 59–85 million reads per sample. The mean per-base Phred-score exceeded 35. Preprocessing included adapter trimming and quality filtering with trim-galore, followed by alignment to GRCm39 using Salmon (v1.5.0) with a decoy-aware transcriptome index (k=31) (58). Annotation used GENCODE M27, with transcript-to-gene conversion via tximport (v1.28.0). DESeq2 (v1.40.24) handled normalization and differential expression analysis. PCA was performed using prcomp on variance-stabilized gene expression. Heatmaps were generated with ggplot2 (v3.5.0) and ComplexHeatmap (v2.16.0). GO clustering used clusterProfiler (v4.2.0), and Gene Set Variation Analysis (GSVA v1.50) utilized MSigDB v7.5.1. A unique CD49a^+^NK gene signature was extracted from metastatic liver samples and applied to single-cell RNA-seq clusters (8).

### scRNA-seq analysis.

scRNA-seq data from PRJNA656253 and GSE164522 were processed using Seurat (v4.0.1) in RStudio (v4.0.2). Low-quality cells (gene count less than 200 or greater than 2,500) and those with more than 10% mitochondrial gene expression were removed. Only NK cells were analyzed in GSE164522. Data normalization and scaling used “SCTransform,” regressing out mitochondrial and cell cycle genes. Highly variable genes (HVG, *n* = 3000) informed PCA, with 20 PCs used for mouse and 30 for human datasets. Graph-based clustering (resolution 0.6 for mouse, 0.3 for human) and UMAP embedding were applied. Differentially expressed genes (DEGs) were identified with “FindAllMarkers” using the Wilcoxon rank sum test (min.pct ≥ 25%, adjusted *P* ≤ 0.05). CD49a^+^NK cluster scoring used “AddModuleScore.” Pseudotime analysis utilized Dynoverse (v0.1.1) with the slingshot algorithm, setting trILC1 as the starting node. Human dataset differential gene expression was assessed with “FindMarkers.”. GO analysis (top 100 genes per NK population) used clusterProfiler (v4.12.0) and msigdbr (v7.5.1) for Homo sapiens categories H, C2, C5, and C7. NK–myeloid interactions were predicted using multinichenetr (v2.0.1, (https://www.biorxiv.org/content/10.1101/2023.06.13.544751v1) with specified parameters (min_cells=5, min_sample_pro*P* = 0.5, fraction_cutoff=0.15, scenario=”lower_DE”).

### Microarray analysis.

Microarray data on CRC-derived liver metastases were obtained from GSE159216 (34). Normalized expression data were retrieved from GEO, and probe IDs were converted to gene symbols using pd.hta.2.0 (v3.12.2), hta20transcriptcluster.db (v8.8.0), and biomaRt (v2.60.0). Gene signature scores were calculated with GSVA using the top 25 NK_CXCR3 markers (by Log_2_ fold change). Multivariate Cox regression analysis, performed in survival (v3.7-0), assessed the gene signature’s median alongside clinical data. Forest plots were generated with survminer (v0.4.9) and sjPlot (v2.8.16).

### Statistics.

Statistical analyses were performed using GraphPad Prism Software. The significance between more than 2 groups was evaluated using 1- or 2-way ANOVA. Comparison between 2 groups were evaluated by paired or unpaired 2-tailed Student’s *t* test. Mann-Whitney test was used in case of non-Gaussian distribution. *P* value less than 0.05 was considered significant. A simple linear regression analysis was performed to evaluate the association between CD49a^+^ NK cells and CXCL9^+^ MHCII^+^F4/80^hi^ macrophages within mouse metastasis. Asterisks denote significant differences.

### Study approval.

Procedures on mice, treatments described, and criteria for euthanasia were approved by the Italian Health Ministry under protocols 727/2019-PR and 136/2024-PR and conformed to guidelines of DL n.26, March 2014.

### Data availability.

Data are available in the Supplemental Material. RNA-seq data can be accessed via NCBI’s GEO repository (accession number GSE266900) at https://www.ncbi.nlm.nih.gov/geo/query/acc.cgi?acc=GSE266900 The mouse and human single-cell sequencing data are available under PRJNA656253 (NCBI Bio Sample) and GSE39583, respectively. Microarray data: GSE159216. Datasets are available from the corresponding author upon request. Values for all data points in graphs are reported in the Supporting Data Values file.

## Author contributions

ER was responsible for planning and conducting most of the experimental work, analyzing and curating the data, and writing the manuscript. CDA and CDC were responsible for conducting experiments. ML was responsible for performing single cell RNA-Seq analysis and transcriptomic analysis of human samples. LT was responsible for conducting some experiments. VL was responsible for supervising and performing bulk RNA-Seq analysis. SG and FS were responsible for performing immunofluorescence experiments. JP was responsible for analyzing bulk RNA-Seq analysis. GP was responsible for performing sorting experiments. AK and UP were responsible for providing mouse strain. CG was responsible for helping in establishment of the metastasis model and revising the manuscript. CAJV was responsible for providing mouse strain and revising the manuscript. CL was responsible for supervising immunofluorescence experiments. SS was responsible for supervising single cell RNA-Seq analysis. GS was responsible for supervising experiments in conditional knock out mice and revising the manuscript. AS was responsible for supervising all the experimental work. GB was responsible for designing research studies, writing the manuscript.

## Supplementary Material

Supplemental data

Supplemental figure 2 data set

Supporting data values

## Figures and Tables

**Table 1:**
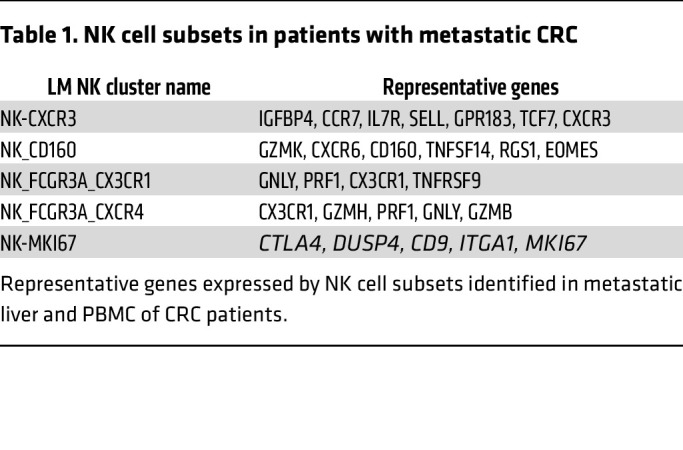
NK cell subsets in patients with metastatic CRC

## Version 1 04/01/2025

In-Press Preview

## Version 2 06/02/2025

Electronic publication
