# Supplementary material for: Cxcr3 promotes protection from colorectal cancer liver metastasis by driving NK cell infiltration and plasticity
Source: J Clin Invest. 2025 Apr 1;135(11):e184036. doi: 10.1172/JCI184036 (PMC12126236; doi:10.1172/JCI184036)

Supplemental Figure Legends

S. Figure 1. Transcriptomic analysis of type 1 ILC in liver metastasis. (A) Gating strategy for cell sorting of ILC1, CD49a$^+$ and CD49a$^-$NK cells from metastasis-free liver (MFL) and liver metastasis (LM) in the MC38 metastasis mouse model. Cells first gated based on forward and side scatter area parameters (FSC-A and SSC-A) were then selected for doublet exclusion (Heights, H versus Area, A). NKp46$^+$ cells were finally collected based on CD49a and CD49b expression levels. **(B)** Volcano plots of differentially expressed genes between LM and MFL NK cells and CD49a$^+$NK cells and MFL or LM CD49a$^-$NK cells. FDR < 0.1 was considered significant. **(C)** Gene Ontology (GO) of upregulated (top) and downregulated (below) genes.

S. Figure 2. MC38-induced liver metastasis microenvironment comprises a variety of cell-state transitions in NK clusters. (A) Top: Umap of cell clusters from analysis of scRNA-seq from (8). Bulk RNAseq was used to produce a unique gene signature for CD49a$^+$NK cells (*1700025G04Rik, Prss16, Tpx2, H2bc23, Cdk6, Plac8, kKif20a, Cenpf, Atad5, Depdc1b, Smtn, Nt5e, 2410022M11Rik, H34, Emb, Tmprss6, 5830408C22Rik, H2aCc10, Spag5, Nsl1, Cxcl10, Sfxn5*) generated by extracting the genes uniquely expressed by CD49a$^+$NK cells from the liver metastatic samples; Violin plots show scoring of clusters for in house signatures. **(B)** Pseudotime analysis on clusters. Schematic of cluster trajectory in pseudotime analysis. **(C)** Heatmap showing top 60 DEGs on cell clusters along with trajectory analysis. **(D)** Most upregulated genes at transition and branching points of pseudotime analysis.

S. Figure 3. Distinctive type 1 ILC markers in healthy liver and MC38-induced metastasis-free liver and liver metastasis. (A) Histogram plots show gMFI ± SEM of CXCR3, CD11b, CD69, CD62L, TIGIT, PD-L1, CD73 and CX_3CR1 expression and mean frequency ± SEM of CD39$^+$ and CXCR6$^+$ cells among ILC1 (blue), CD49a$^+$NK (orange) and CD49a$^-$NK (red) cells in MFL and LM (CXCR3 n=7, **p=0.002 one-way ANOVA in LM,

CD62L n=9 **p=0.003, CD69 n=3 **p=0.0013, CD73 n≥3 *p=0.06, CXCR6 n≥8 ***p=0.0002, one-way ANOVA). **(B)** Representative FACS histogram plots show CD62L, CXCR3, CD73, CD39, TIGIT, CXCR6, KLRG1, CX_3CR1 and CD69 expression on ILC1, $CD49a^+$NK and $CD49a^-$NK cells in healthy liver (HL), MFL and LM. At least three independent experiments were performed. **(C)** Representative histograms of Ki-67 expression and relative mean frequency ± SEM in ILC1, $CD49a^+$NK and $CD49a^-$NK from MFL and LM (n=4, *p=0.01, **p=0.009, one-way ANOVA). **(D)** Histogram shows mean frequency ± SEM of $BrdU^+$ cells among ILC1, $CD49a^+$NK and $CD49a^-$NK in LM upon *in vivo* staining with BrdU (n=3).

S. Figure 4. CXCR3 identifies a liver metastasis-associated NK cluster in human patients. **(A)** Umap of NK cell clusters in MFL, LM and peripheral blood mononuclear cells (PBMCs) from a dataset of human liver metastasis colorectal cancer patients (32). **(B)** Heatmap of top 10 DEGs in NK cell clusters.

S. Figure 5. Expression of effector molecules and killing capacity of type 1 ILC in liver metastasis. **(A)** Representative pictures of metastatic livers from MC38- and SL4-injected mice**.** **(B)** Quantification by ELISA of IFN-γ concentration (pg/10^6 cells) in supernatants from sorted splenic NK (black), HL NK (grey), MFL NK (green), LM $CD49a^-$NK and LM $CD49a^+$NK cells (*p=0.04, ***p=0.0002, one-way ANOVA). **(C)** Top: representative FACS histogram plots of Perforin, Granzyme B (GzmB), Granzyme A (GzmA) and Granzyme C (GzmC) expression on MFL ILC1 and NK cells and on LM ILC1, $CD49a^+$NK and $CD49a^-$NK cells. Dotted line represents FMO staining control. Below: histogram plots show mean frequency ± SEM of $Perforin^+$, Granzyme B^+, Granzyme A^+ and Granzyme C^+ cells on MFL and LM ILC1, $CD49a^+$NK and $CD49a^-$NK cells. Three independent experiments with 4 mice per group were performed. For Perforin expression, n=4 (GzmA n ≥ 8 **p=0.002, GzmC n ≥14

****p<0.0001, one-way ANOVA). **(D)** Histograms show comparison of frequency of granzyme A, B and C expression on LM ILC1, CD49a$^+$NK and CD49a$^-$NK from MC38- and SL4-derived liver metastasis (MC38 n=5, SL4 n=3, **p=0.001, **p=0.004, ***p=0.0002, one way ANOVA).

S. Figure 6. Myeloid cell distribution and function in metastatic liver. (A) Gating strategy of myeloid cells in LM. **(B)** Histogram plots show frequency of dendritic cells (Total DC) (CD11c$^+$MHCII$^+$), cDC1 (CD11c$^+$MHCII$^+$CD11b$^-$Xcr1$^+$) and cDC2 (CD11c$^+$MHCII$^+$CD11b$^+$) among live lin$^-$CD45$^+$ cells (**p=0.001, one-way ANOVA) **(C)** TGF-β concentration (pg/ml) in supernatants of LM macrophages, MC38 and SL4 cells. Medium was used as control. **(D)** Representative FACS histograms of Arg1, MMR, PD-L1, LAP-1, MHCII and TREM2 expression on F4/80^{high} and F4/80^{int} LM macrophages. Light grey histograms show isotypic staining control, dark grey histograms show FMO control in HL, MFL and LM. **(E)** Histograms show gMFI ± SEM of Ly6C, CD11c, CD11b, CX_3CR1 and frequency of Tim4$^+$ cells in LM F4/80^{int} and F4/80^{high} macrophages (n>4, Ly6C **p=0.002, CD11c **p=0.04, CX_3CR1 **p=0.002, Tim4 **p=0.02, two-tailed Student's *t* test). **(F)** Frequency ± SEM of CXCL9$^+$ cells among F4/80^{int} and F4/80^{high} macrophages and dendritic cells from LM (n>3 in three independent experiments, **p=0.07, **p=0.01, one-way ANOVA).

S Figure 7. Macrophages from MC38-induced metastasis contribute to CD49a$^+$ NK cell generation. (A) Left: contour plots show F4/80 and CD11b expression on purified macrophages from LM and MFL. Representative contour plots and scatter plots show gMFI ± SEM of Tim4, Ly6C and CD115 on F4/80^{high} (green) and F4/80^{int} (grey) subsets of macrophages from LM and MFL (Tim4 LM **p=0.001, MFL *p=0.02, Ly6C LM **p=0.001, MFL *p=0.01, CD115 MFL *p=0.05, two-tailed Student's *t* test). **(B)** Fold change (F.C.) of CD49a$^+$NK cell frequency upon 72 hours of co-culture with MC38-derived MAMs in presence

or absence of cell-cell contact (n=3, *p=0.02, two-tailed Student's *t* test). **(C)** Frequency ± SEM of CD107a$^+$ expression induced by anti-NK1.1 mAb stimulation on NK cells cultured with IL-15 alone (grey) or in combination with TGF-β (orange) or upon co-culture with LM macrophages (red) for 72 hours (n≥3, **p=0.003, ***p=0.0006, one-way ANOVA). **(D)** Frequency ± SEM of BrdU$^+$NK cells upon 72 hours with IL-15 (10ng/ml) alone or in co-culture with MC38- or SL4-derived LM macrophages (****p<0.0001, one-way ANOVA). From panel (A) to (D) at least three independent experiments are shown. **(E)** Cell interaction analysis of macrophage and NK cell clusters in human liver metastasis from analysis of (32).

S Figure 8. MC38-derived MAMs, but not mono-macrophages, contribute to CD49a$^+$ NK cell generation. **(A)** Representative picture of metastatic livers from Rat IgG- and anti-CSF1R-treated mice. **(B)** Hematoxylin-eosin staining of liver sections from Rat IgG- and anti-CSF1R-treated mice and relative quantification. Histogram plot shows metastasis weight (gr) in Rat IgG- and anti-CSF1R-treated mice. Mean ± SEM of two independent experiments. **(C)** Top: representative contour plots of F4/80^{int} and F4/80^{high} macrophages in Rat IgG- and anti-CSF1R-treated LM. Below: histogram plots show frequency ± SEM of F4/80^{int} and F4/80^{high} macrophages in LM (n=9 ****p<0.0001, two-tailed Student's *t* test). **(D)** Violin plot shows concentration of CXCL10 in LM from Rat IgG- and anti-CSF1R-treated tumor-bearing mice. Histogram plots show gMFI ± SEM of CXCL9 and LAP-1 in leftover macrophages from Rat IgG- and anti-CSF1R-derived LM (n>2, *p=0.02, two-tailed Student's *t* test). **(E)** Mean frequency ± SEM of monocytes, macrophages and dendritic cells in bone marrow from Rat IgG- or anti-CSF1R-treated tumor-bearing mice (n=4). **(F)** Representative pictures of metastatic livers in Rat IgG- and MC-21-treated mice. Histogram plots show number of metastasis, % of inhibition ± SEM of F4/80^{int} and F4/80^{high} macrophage frequency in LM of Rat IgG- and MC-21-treated tumor-bearing mice (n>5, **p=0.07, two-tailed Student's *t* test).

S. Figure 9. Immune cell distribution in the liver from Ncr1$^{\Delta Cxcr3}$ mice. (A) Left: schematic representation of mouse breeding to obtain Ncr1$^{\Delta Cxcr3}$; right: representative FACS histogram plots of CXCR3 expression on ILC1, NK cells and CD3^{+} cells from liver of Cxcr3$^{fl/fl}$ and Ncr1$^{\Delta Cxcr3}$ mice. **(B)** Histogram plots show frequency ± SEM of liver type 1 ILCs (live lin- CD45^{+}NK1.1^{+}NKp46^{+}), NK cells and ILC1 and of Kupffer cells, dendritic cells (DCs) and macrophages in Cxcr3$^{fl/fl}$ and Ncr1$^{\Delta Cxcr3}$ mice at steady state (n=3/group).

Supplemental Figure 1

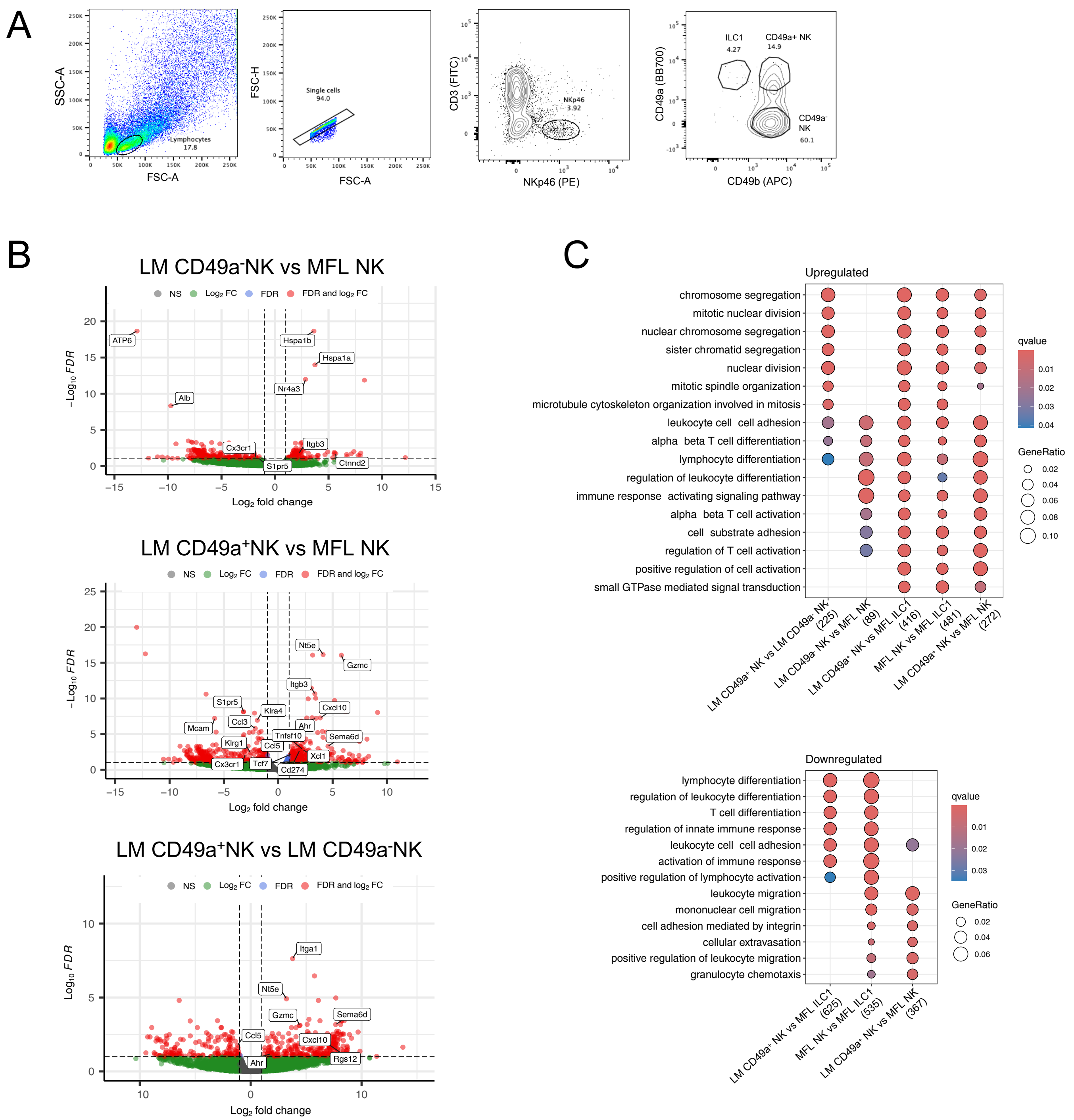

Supplemental Figure 2

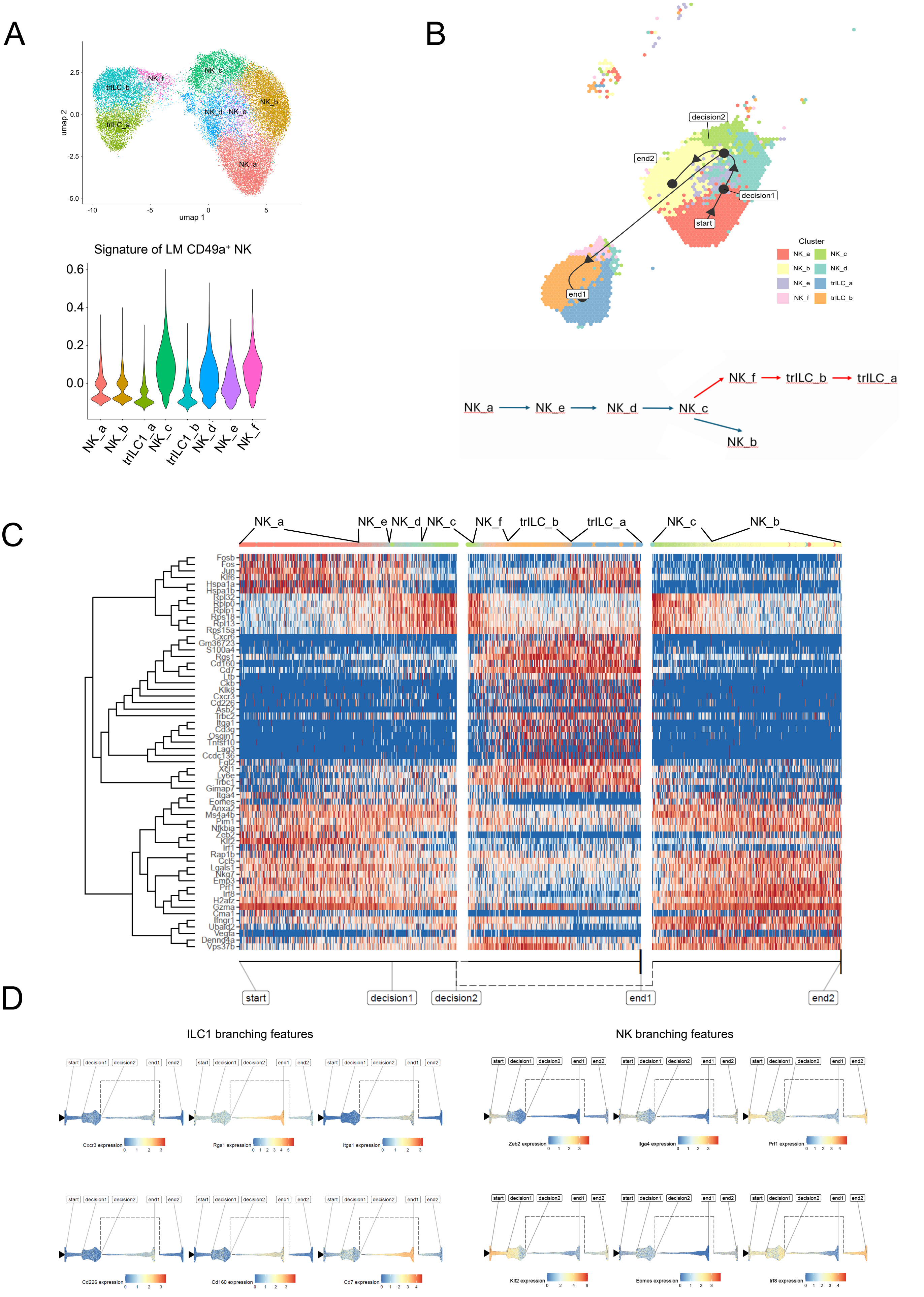

Supplemental Figure 3

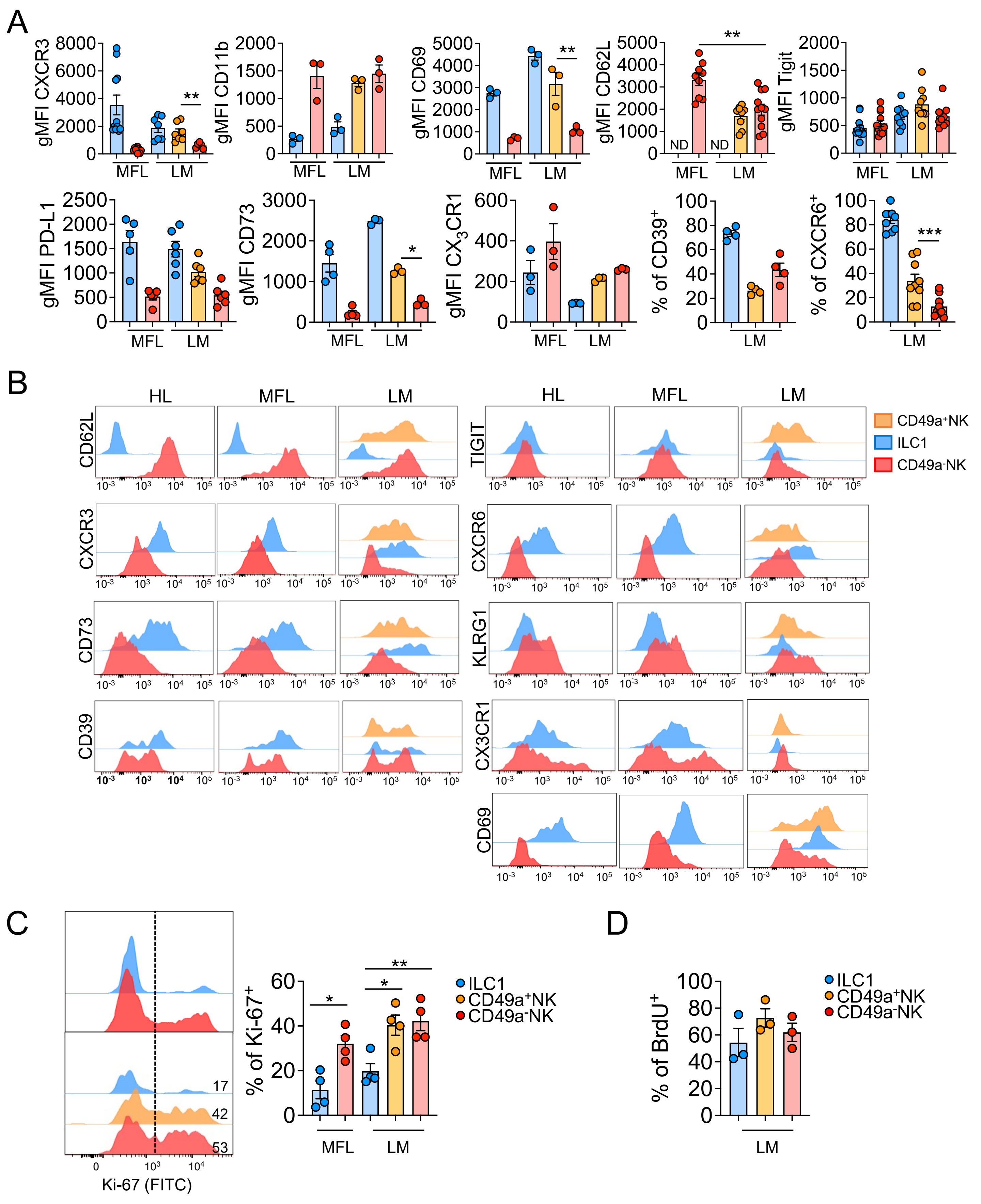

Supplemental Figure 4

A

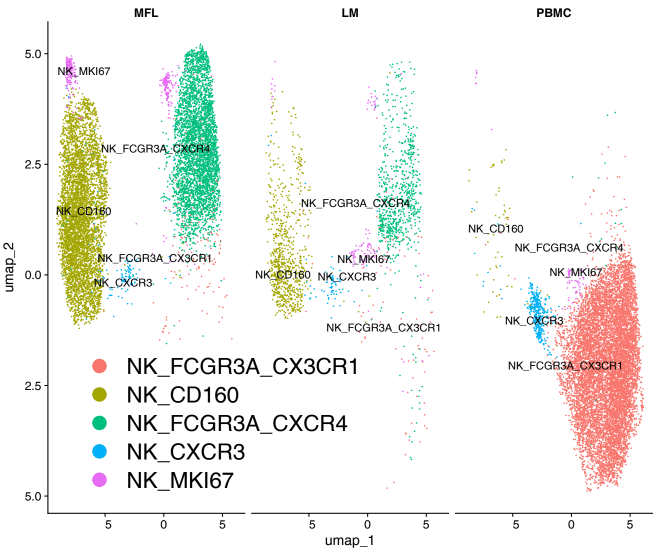

B

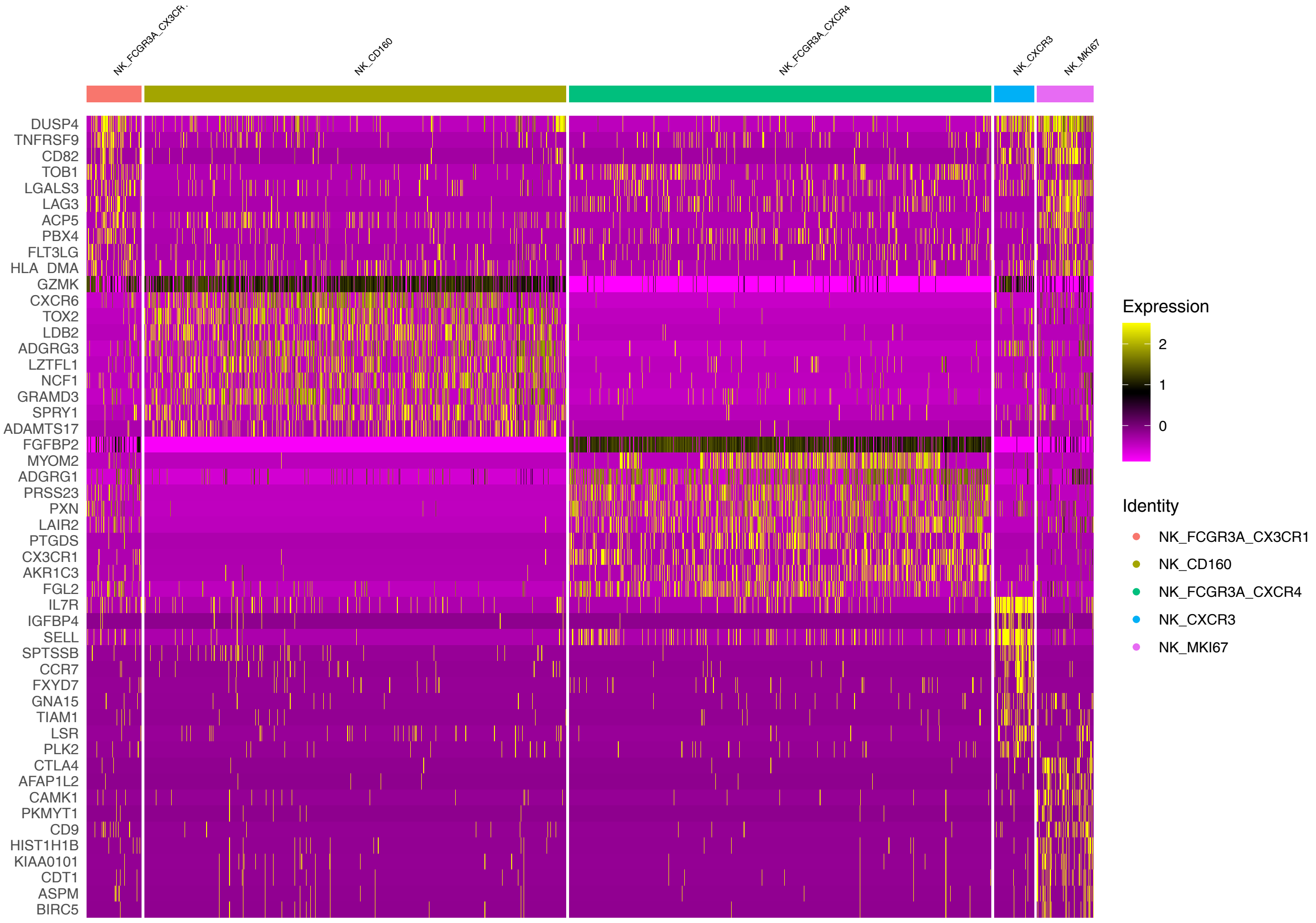

Supplemental Figure 5

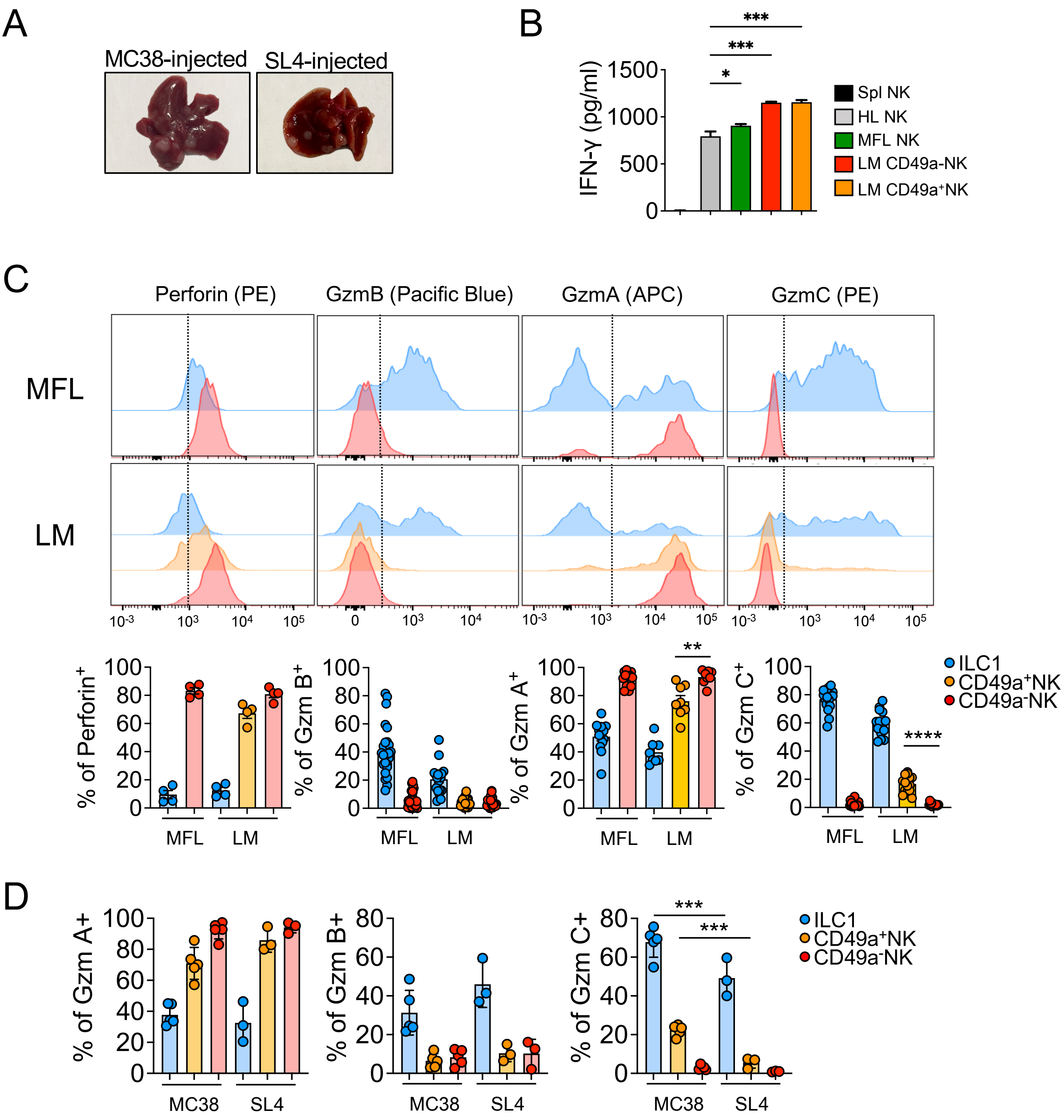

Supplemental Figure 6

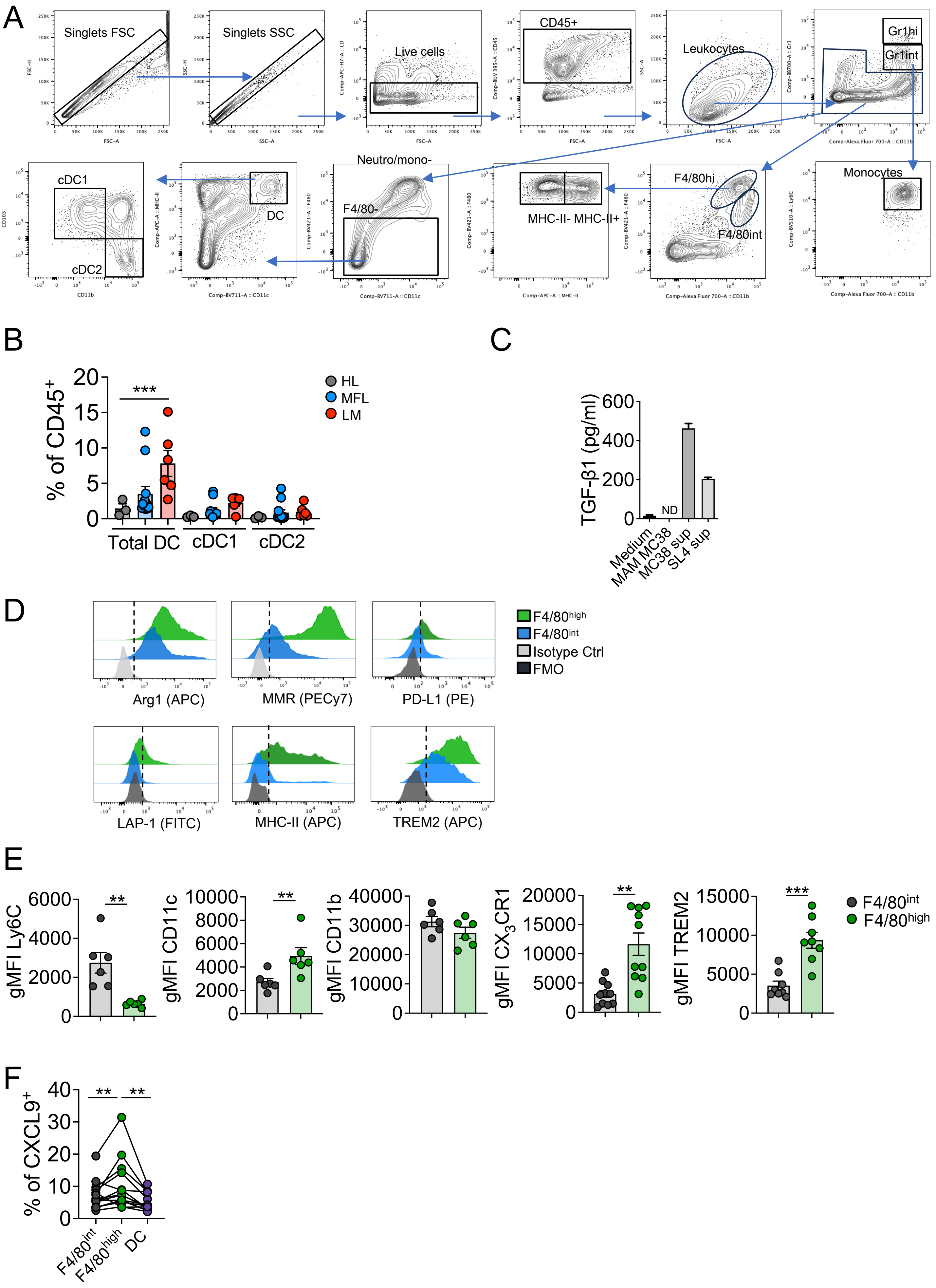

Supplemental Figure 7

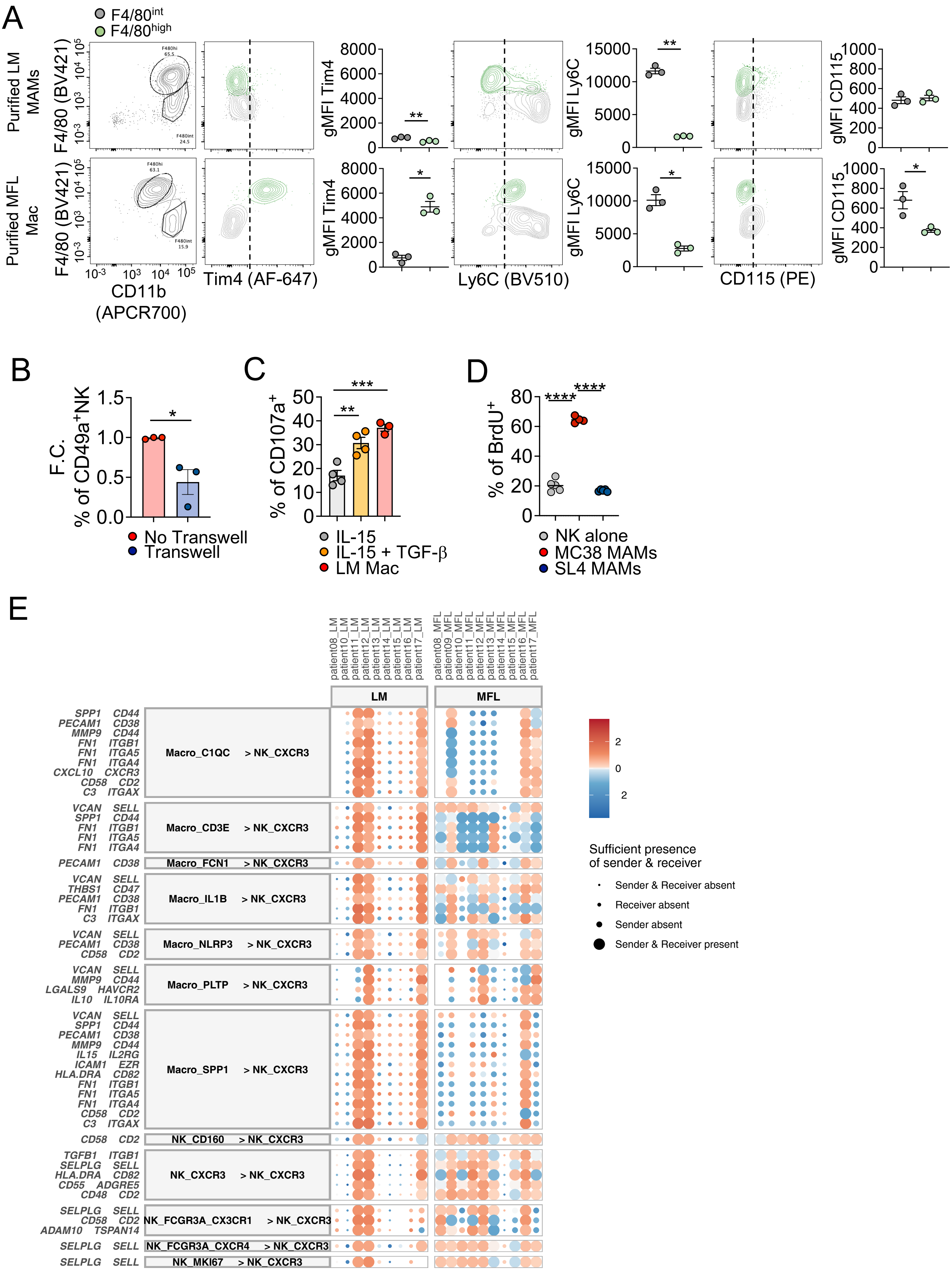

Supplemental Figure 8

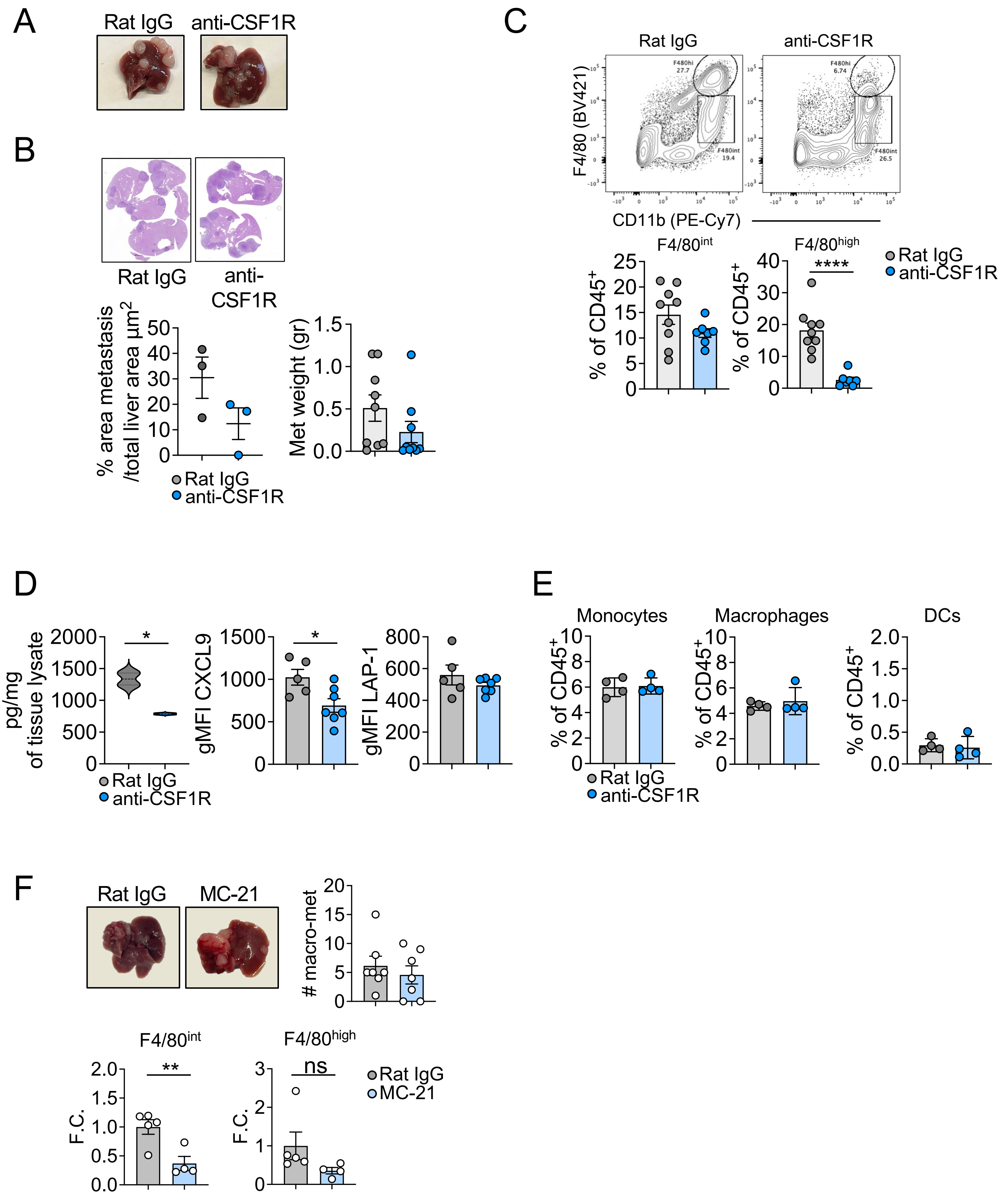

Supplemental Figure 9

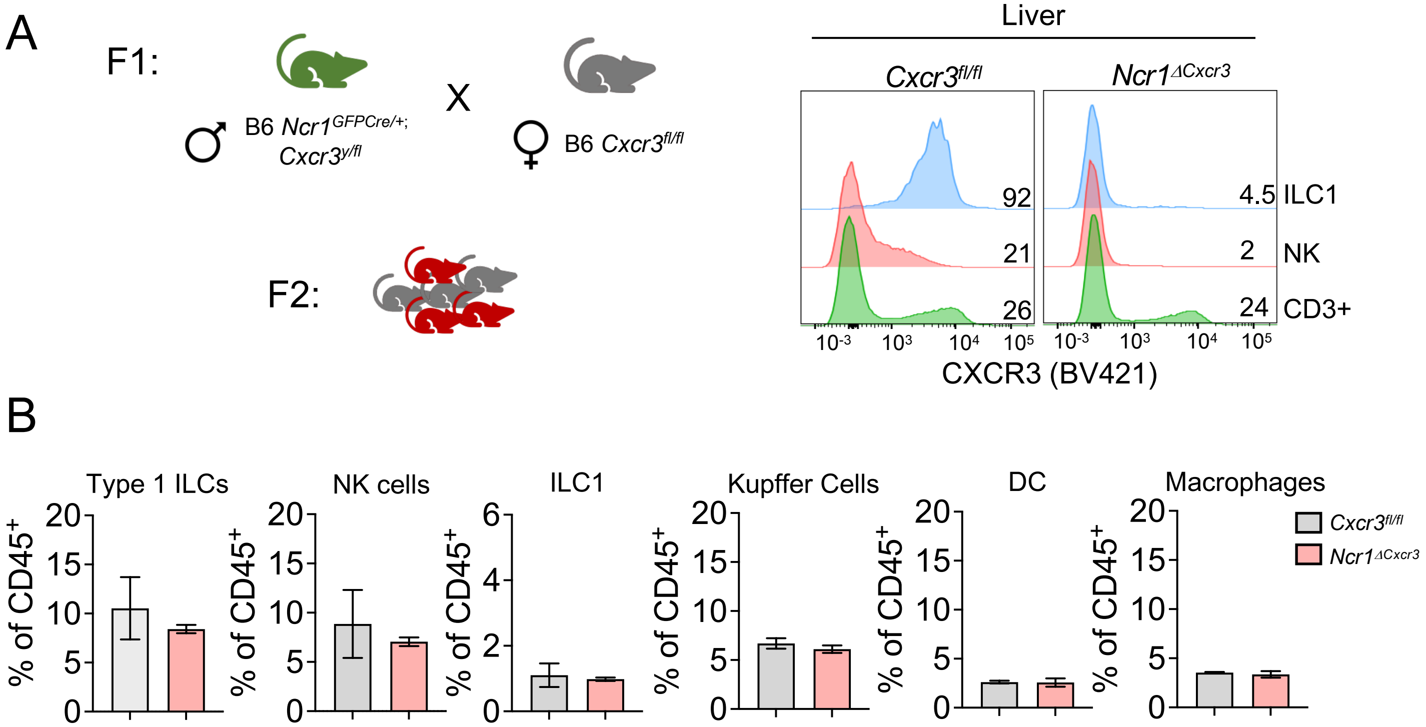

Supplement: Supplemental data [file jci-135-184036-s172.pdf]
